# Engineering Dual Active Sites and Defect Structure in Nanozymes to Reprogram Jawbone Microenvironment for Osteoradionecrosis Therapy

**DOI:** 10.1002/advs.202413215

**Published:** 2024-12-17

**Authors:** Zheng Cheng, Yuchen Wang, Haobo Lin, Ziyu Chen, Ran Qin, Tianxiao Wang, Hang Xu, Yifei Du, Hua Yuan, Yongchu Pan, Huijun Jiang, Xinquan Jiang, Jiandong Jiang, Fan Wu, Yuli Wang

**Affiliations:** ^1^ Department of Oral and Maxillofacial Surgery The Affiliated Stomatological Hospital of Nanjing Medical University State Key Laboratory Cultivation Base of Research Prevention and Treatment for Oral Diseases Jiangsu Province Engineering Research Centre of Stomatological Translational Medicine Nanjing Medical University Nanjing Jiangsu 210029 China; ^2^ Medical Basic Research Innovation Centre for Cardiovascular and Cerebrovascular Diseases Ministry of Education International Joint Laboratory for Drug Target of Critical Illnesses School of Pharmacy Nanjing Medical University Nanjing Jiangsu 211166 China; ^3^ State Key Laboratory of Systems Medicine for Cancer Shanghai Cancer Institute Renji Hospital Affiliated to Shanghai Jiao Tong University School of Medicine Shanghai 200120 China; ^4^ Department of Orthodontic The Affiliated Stomatological Hospital of Nanjing Medical University State Key Laboratory Cultivation Base of Research Prevention and Treatment for Oral Diseases Jiangsu Province Engineering Research Centre of Stomatological Translational Medicine Nanjing Medical University Nanjing Jiangsu 210029 China; ^5^ Department of Prosthodontics Shanghai Ninth People's Hospital Shanghai Jiao Tong University School of Medicine College of Stomatology Shanghai Jiao Tong University Shanghai Engineering Research Center of Advanced Dental Technology and Materials National Center for Stomatology National Clinical Research Center for Oral Diseases Shanghai Key Laboratory of Stomatology Shanghai Research Institute of Stomatology No. 639 Zhizaoju Road Shanghai 200011 China; ^6^ Institute of Medicinal Biotechnology Chinese Academy of Medical Sciences & Peking Union Medical College Beijing 100050 China

**Keywords:** enzyme‐like performance, mitophagy, nanozymes, osteoradionecrosis of the jaw, reactive oxygen and nitrogen species

## Abstract

Four to eight percent of patients with head and neck cancer will develop osteoradionecrosis of the jaw (ORNJ) after radiotherapy. Various radiation‐induced tissue injuries are associated with reactive oxygen and nitrogen species (RONS) overproduction. Herein, Fe doping is used in VO_x_ (Fe‐VO_x_) nanozymes with multienzyme activities for ORNJ treatment via RONS scavenging. Fe doping can induce structure reconstruction of nanozymes with abundant defect production, including Fe substitution and oxygen vacancies (OVs), which markedly increased multiple enzyme‐mimicking activity. Catalase (CAT), superoxide dismutase (SOD), and glutathione peroxidase (GPx) enzyme‐like performance of Fe‐VO_x_ can effectively reprogram jawbone microenvironment to restore mitochondrial dysfunction and enhance mitophagy. Moreover, the surface plasmon resonance (SPR) effect of Fe‐VO_x_ made it a good photothermal nanoagents for inhibiting jaw infection. Thus, this work demonstrated that Fe‐VO_x_ nanozymes can efficiently scavenge RONS, activate mitophagy, and inhibit bacteria, which is potential for ORNJ treatment.

## Graphical abstract

CAT, SOD, and GPx enzyme‐like performance of Fe‐VO_x_ could effectively reprogram jawbone microenvironment to efficiently scavenge RONS and activate mitophagy, which is potential for ORNJ treatment.

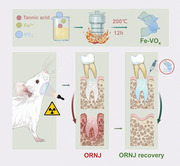

## 1 Introduction

Radiotherapy is a standard therapeutic strategy for head and neck cancer,^[^ ^1^ ^]^ and ≈66% of patients with malignancies located in the head and neck regions receive radiotherapy.^[^ ^2^ ^]^ Radiotherapy works as a double‐edged sword which eliminates malignant tumor cells but also impairs the vitality of normal tissue. Osteoradionecrosis of the jaw (ORNJ) is one of the most severe complications after radiotherapy without effective medicine, and the incidence of ORNJ was 4–8% in patients undergoing head and neck radiation.^[^ ^3^ ^]^ Hypoxic‐hypocellular‐hypovascular, and non‐regulated cellular differentiation, especially the impaired osteogenic differentiation of mesenchymal stem cells (MSCs), are the primary mainstream theories involved in the pathogenesis.^[^ ^4^ ^]^ Although current measures include hydrogen peroxide solution irrigation, systemic antibiotics, and hyperbaric oxygen permit the control of localized lesions,^[^ ^5^ ^]^ however, extended resection of the jaw is essential for refractory and advanced stage ORNJ. Additionally, operative treatment is often accompanied by other complication, such as surgical trauma, pain, and microvascular osteomyocutaneous free flap necrosis.^[^ ^6^ ^]^ Therefore, it is urgently needed to elucidate the mechanisms of ORNJ and design alternative therapeutic strategies for precise treatment.

Various radiation‐induced tissue injuries are unwaveringly associated with reactive oxygen and nitrogen species (RONS) overproduction.^[^ ^7^ ^]^ RONS, such as H_2_O_2_, •O_2_^−^, •OH, •NO in mitochondrion,^[^ ^8^ ^]^ is a vital part of host immune defense system. RONS is important for eliminating pathogens infection and the amplification of inflammatory cytokine release.^[^ ^9^ ^]^ However, excess RONS accumulation caused by radiation leads to inflammatory/anti‐inflammatory imbalance, and systemic dysfunction.^[^ ^10^ ^]^ Moreover, the high levels of RONS also can induce mitochondrial dysfunction further suppress mitophagy, which disrupts mitochondrial homeostasis and aggravates in cell death.^[^ ^11^ ^]^ Mitophagy, a type of selective specific autophagy, is response for degrading dysfunctional mitochondria and essential for cellular homeostasis. Mitochondrial injury mediated by RONS causes elevated oxidative damage, and mitophagy prevents excessive RONS accumulation, activates the mitochondrial apoptotic cascade, and ensure the quality control of mitochondria.^[^ ^12^ ^]^ From this perspective, reprogramming the RONS level and reversing oxidative/anti‐oxidative imbalance to repair mitochondrial dysfunction and enhance mitophagy in jawbone microenvironment are expected to alleviate ORNJ. Inspired by enzyme‐like catalysis,^[^ ^13^ ^]^ lots of enzyme‐mimicking nanomaterials have been developed for oxidative stress‐induced diseases, such as metal‐based nanozymes,^[^ ^14^ ^]^ metal oxide‐based nanozymes,^[^ ^15^ ^]^ and carbon‐based nanozymes.^[^ ^16^ ^]^ The special structure and composition of these nanozymes make them possess antioxidative enzyme‐like activity. Eliminating RONS by nanozymes opens up a new way for ORNJ catalytic therapy. Although adjusting RNOS level of jawbone microenvironment is a promising therapeutic strategy, achieving an ideal enzyme‐like catalytic effect is still a challenge. It has been demonstrated that the defect of nanozymes play a key role in improving enzyme‐like activity.^[^ ^17^ ^]^ The defective formation of nanomaterials can markedly adjust the structure reconstruction and chemical properties, which facilitate the generation of new physicochemical property or robust joint effects to improve the nanozymes performance.^[^ ^18^ ^]^ Therefore, appropriate defect structure customization can develop nanozymes with strong enzyme‐mimicking functions and tunable properties,^[^ ^19^ ^]^ such as, WO_x_ nanoribbons,^[^ ^20^ ^]^ CeO_2_ nanoparticles,^[^ ^21^ ^]^ and MoO_3−x_ nanodots.^[^ ^22^ ^]^ However, the enzyme‐like activity of oxygen vacancies (OVs) defective nanozymes is still restrained by its single metallic element composition with the relatively single catalytic route. The excellent charge transfer effect of different metal elements and potential combined effects, which make foreign metal‐doped nanozymes with better chemical stability and activity compared to pure metal oxide nanozymes.^[^ ^23^ ^]^ Multi‐metal elements endow plenty of active sites for catalysis, which can enhance enzyme‐like activity via the mutual effect of various element sites of nanozymes. Coincidentally, metal heteroatom doping is an effective method to construct defects in nanozymes.^[^ ^19^ ^]^ The introduced dopant atoms can be served as substitution of existing atoms on the lattice or interstitial positions.

**Figure 1:**
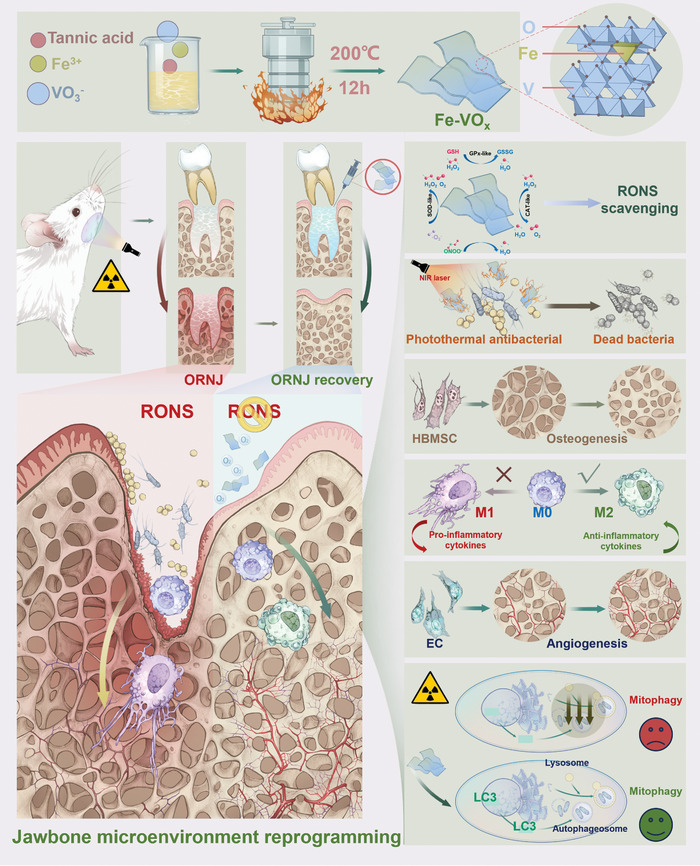
Schematic illustration of the synthetic method and therapeutic mechanisms of ORNJ. Fe‐VO_x_ nanozymes can treat ORNJ via reprogramming jawbone microenvironment.

Herein, we developed Fe‐doped vanadium oxide (Fe‐VO_x_) nanozymes for ORNJ therapy (**Figure** **1**). Fe‐VO_x_ can synchronously enrich OVs defect and Fe substitution. The surface plasmon resonance (SPR) effect caused by defect further equips Fe‐VO_x_ with an excellent photothermal performance in near‐infrared (NIR) region, which can convert NIR into heat, for antibacterial photothermal therapy (PTT) of jaw. Benefiting from the formation of synergistic active sites by interaction of V and Fe, Fe‐VO_x_ nanozymes possesses an excellent tri‐enzyme‐like activity [catalase (CAT), superoxide dismutase (SOD), glutathione peroxidase (GPx)]. Density functional theory (DFT) calculations systematically demonstrate that the efficient RNOS scavenging via joint effect of dual active sites. Fe‐VO_x_‐induced RONS scavenging can increase mitophagy of macrophage, endothelial cell (EC), and human bone marrow mesenchymal stem cells (HBMSC) for enhancing anti‐inflammatory properties, blood vessels forming, and osteogenic differentiation, respectively. Our work not only developed nanozymes with unexpected catalytic effect but also provides a next‐generation therapeutic strategy of ORNJ.

## 2 Results and Discussion

### 2.1 Synthesis and Characterization of Fe‐VO_x_

**Figure 2:**
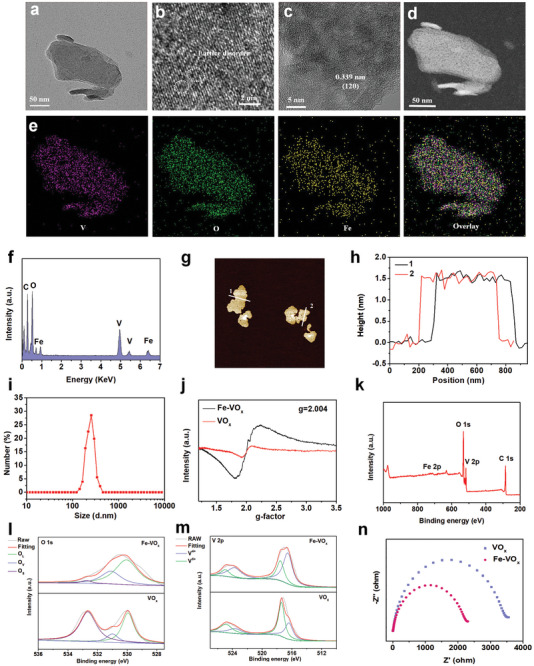
Characterization of Fe‐VO_x_. a) TEM images of Fe‐VO_x_ nanozymes. b) HRTEM image of the Fe‐VO_x_ nanozymes. c) HRTEM image of the Fe‐VO_x_ with clear lattice fringes. d) HAADF‐STEM image of the Fe‐VO_x_ nanozyme and e) corresponding EDS mapping. f) EDS spectrum of Fe‐VO_x_ nanozymes. g) AFM image of Fe‐VO_x_ and (h) the corresponding height profile. i) DLS measurement of Fe‐VO_x_ nanozymes. j) ESR spectra of Fe‐VO_x_ and VO_x_ samples. k) XPS data of Fe‐VO_x_. l) O 1s XPS spectra of Fe‐VO_x_ and VO_x_ samples. O_L_, O_V_, and O_S_ are associated with lattice oxygen, OVs, and surface‐absorbed oxygen, respectively. m) V 2p XPS spectra of Fe‐VO_x_ and VO_x_. n) EIS Nyquist plots of Fe‐VO_x_ and VO_x_.

The preparation of VO_x_ and Fe‐VO_x_ nanozymes were performed by exploiting metal‐polyphenol coordination tactics (Figure S1, Supporting Information). As shown in **Figures** **2**a and S2 (Supporting Information), transmission electron microscopy (TEM) image demonstrated that both of Fe‐VO_x_ and VO_x_ with 2D flake structures. In high‐resolution TEM (HRTEM) image (Figure 2b), apparent lattice disorder and dislocation could be found in Fe‐VO_x_ nanozymes, which was due to defect formation. Besides, HRTEM image and X‐ray diffraction (XRD) patter both indicated Fe‐VO_x_ with crystal structure (Figure 2c; Figure S3, Supporting Information). The diffraction peaks were in line with VO_2_ (JCPDS No. 25‐1003), and no diffraction patterns related to Fe can be observed, indicating that the existence of Fe is substitutional form. The homogeneous V, O, and Fe element distributions of Fe‐VO_x_ can be verified by the high‐angle annular dark‐field scanning TEM (HAADF‐STEM) and energy‐dispersive X‐ray spectroscopy (EDS) elemental mapping profiles (Figure 2d–f). EDS element mapping also demonstrated the uniform distribution of V, O in VO_x_ (Figure S4, Supporting Information). The atom ratio of Fe and V was 1: 9.7 in Fe‐VO_x_ nanozymes by using inductively coupled plasma mass spectroscopy (ICP‐MS) measurement. As revealed in the atomic force microscopy (AFM) image illustrated that the thickness of Fe‐VO_x_ is ≈1.5 nm (Figure 2g,h). Dynamic light scattering (DLS) measurements indicated that both of Fe‐VO_x_ and VO_x_ nanozymes with uniform size distributions (Figure 2i; Figure S5, Supporting Information). Fe substitution can easily induce structure reconstruction and remove oxygen atom of oxide lattice. Electron spin resonance (ESR) spectra was used to characterize such inference. As shown in Figure 2j, for Fe‐VO_x_ nanozymes, an obvious resonance signal at *g* = 2.004 could be observed, which was due to the electrons trapped of OVs.^[^ ^24^ ^]^ In contrast, for VO_x_, a ESR weak signal could be found. The element composition of Fe‐VO_x_ was detected by X‐ray photoelectron spectroscopy (XPS). The characteristic peak of Fe of Fe‐VO_x_ could be observed compared with VO_x_ (Figure 2k; Figure S6a, Supporting Information), indicating Fe element in Fe‐VO_x_. High‐resolution O 1s spectra was used to judge OVs concentrations. As shown in Figure 2l, the OVs content of Fe‐VO_x_ nanozymes was calculated as 29.0% which is higher than VO_x_ (10%), thus verifying that Fe dope increase OVs formation. XPS spectra also confirmed the mixed‐valence states in Fe‐VO_x_, which proved that the binding energies of V 2p_3/2_ and V 2p_1/2_ of V^4+/5+^ in Fe‐VO_x_ were positively shifted, compared with VO_x_ (Figure 2m; Figure S6b, Supporting Information). These effects illustrated the generation of electron transfers from V to Fe, which were in line with electrochemical impedance decline after Fe doping.^[^ ^25^ ^]^ As shown in Figure 2n, a smaller arc radius of Fe‐VO_x_ in the electrochemical impedance spectroscopy (EIS) Nyquist plots. Thus, Fe substitution could speed electron transfer and obtain a higher active site utilization efficiency during whole enzyme‐mimicking catalysis process.^[^ ^26^ ^]^

### 2.2 RONS Scavenging of Fe‐VO_x_

**Figure 3:**
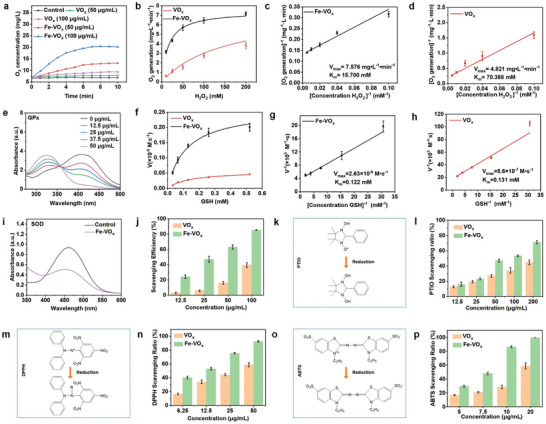
Catalytic performance of Fe‐VO_x_. a) O_2_ concentration of H_2_O_2_ solution with different samples. b) Steady‐state kinetics analysis of Fe‐VO_x_ and VO_x_. c,d) Lineweaver‐Burk plots of CAT‐like activity of Fe‐VO_x_ and VO_x_. e) DTNB absorption with nanozymes of different concentrations. f) Steady‐state kinetics analysis of Fe‐VO_x_ and VO_x_ against GSH substrate. g,h) Corresponding Lineweaver‐Burk plots of GPx‐like activity of Fe‐VO_x_ and VO_x_ against the variation of GSH concentration. i) SOD‐like property of Fe‐VO_x_ for scavenging of •O_2_^−^. j) SOD‐like activity of nanozymes at different concentrations. k) Mechanism of PTIO scavenging assays. l) PTIO scavenging ratio of Fe‐VO_x_ and VO_x_. m) Mechanism of DPPH scavenging assays. n) DPPH scavenging ratio of Fe‐VO_x_ and VO_x_. k) Mechanism of ABTS scavenging assays. l) ABTS scavenging ratio of Fe‐VO_x_ and VO_x_.

The multienzyme‐like activities of Fe‐VO_x_ for reactive oxygen species (ROS) scavenging were systematically investigated. Nanomaterials with CAT‐like activity can break down H_2_O_2_ into H_2_O and O_2_,^[^ ^27^ ^]^ so the change of dissolved O_2_ concentration can be used to measure CAT‐like activity. As shown in **Figure** **3**a, after adding Fe‐VO_x_, a remarkable O_2_ concentration increase compared to the other groups. Besides, obvious bubble generation could be observed after Fe‐VO_x_ mixed with H_2_O_2_ (Figure S7, Supporting Information), reflecting Fe‐VO_x_ with excellent CAT‐like activity. VO_x_ also possessed CAT‐like activity, but its catalytic efficiency was lower than Fe‐VO_x_. Moreover, kinetic investigation indicated that Fe‐VO_x_ had a higher maximum velocity (V_max_ = 7.576 mg•L^−1^•min^−1^) and a lower Michaelis‐Menten constant (K_m_ = 15.700 mm) compared with VO_x_ (V_max_ = 4.821 mg•L^−1^•min^−1^, K_m_ = 70.368 mm) (Figure 3b–d). Therefore, these results confirmed Fe doping increase CAT‐like activity. The combination of GPx and glutathione (GSH) can efficiently change H_2_O_2_ into H_2_O against ROS.^[^ ^28^ ^]^ GPx‐like activity was investigated by using 5,5‐dithiobis (2‐nitrobenzoic acid) (DTNB). The absorbance at 412 nm declined with increasing Fe‐VO_x_ concentration (Figure 3e), indicating a strong GPx‐mimicking capability. Michaelis‐Menten kinetic assessment illustrated that the V_max_ of Fe‐VO_x_ (2.63 × 10^−6^ M•s^−1^) was 4.7 times higher than VO_x_ (5.6 × 10^−7^ M•s^−1^) (Figure 3f–h). Moreover, the K_m_ of Fe‐VO_x_ (0.122 mm) was lower than VO_x_ (0.131 mm). These results revealed that Fe substitution increase the GPx‐like activity of nanozymes. SOD, a conventional antioxidase of cellular defense combatting ROS, can convert •O_2_^−^ into H_2_O_2_ and O_2_. SOD‐like activity can be accessed by using WST‐1, which can react with •O_2_^−^ to generate formazan with the appearance 450 nm characteristic absorbance.^[^ ^29^ ^]^ As shown in Figure 3i, Fe‐VO_x_ had an excellent •O_2_^−^ scavenging effect, manifesting prominent SOD‐like activity (Figure 3j). The scavenging efficiency of Fe‐VO_x_ was superior to that of VO_x_ nanozymes. All these results confirmed that Fe substitution increase multienzyme‐like activity. Besides, Fe‐VO_x_ also had better multienzyme activities than iron oxide. The generated O_2_ concentration of Fe‐VO_x_ was much higher than that of iron oxide group (Figure S8a, Supporting Information), demonstrating that Fe‐VO_x_ exhibited outstanding catalytic decomposition of H_2_O_2_ with high CAT activity. The DTNB absorbance at 412 nm of Fe‐VO_x_ was lower than iron oxide group (Figure S8b, Supporting Information), indicating Fe‐VO_x_ with better GPx activity. Fe‐VO_x_ displayed much better •O_2_^−^ eliminating efficiency superior to iron oxide (Figure S8c, Supporting Information), demonstrating that Fe‐VO_x_ with a better SOD‐like activity.

ESR spectroscopy was further to investigate ROS elimination. As shown in Figure S9 (Supporting Information), ESR signal of •OH and •O_2_^−^ were both decreased, after the addition of Fe‐VO_x_. Next, 2‐phenyl‐4,4,5,5‐tetramethylimidazoline‐1‐oxyl 3‐oxide radical (PTIO) was further used to assess antioxidant ability of Fe‐VO_x_ (Figure 3k). After PTIO incubated with Fe‐VO_x_ for 120 min, the characteristic absorption at 557 nm was markedly decreased (Figure S10, Supporting Information), indicating Fe‐VO_x_ had an excellent PTIO scavenging ability. Compared with VO_x_, Fe‐VO_x_ had a better PTIO scavenging ability (Figure 3l).

As shown in Figure 3m,o, two representative nitrogen free radicals, 2,2‐diphenyl‐1‐picrylhydrazyl radical (DPPH•) and 2,2′‐azino‐bis(3‐ethylbenzthiazoline‐6‐sulfonic acid) radical ion (ABTS^•+^) were utilized to investigate reactive nitrogen species (RNS) scavenging efficiency of nanozymes.^[^ ^21^ ^]^ The characteristic absorption peak at 517 nm of DPPH can be used to judge its reduction.^[^ ^21^ ^]^ After Fe‐VO_x_ reacted with DPPH, the absorption peak was observably declined (Figure S11, Supporting Information), and increasing Fe‐VO_x_ concentration can facilitate DPPH elimination. Moreover, Fe‐VO_x_ showed a higher scavenging efficiency than VO_x_ (Figure 3n), demonstrating that Fe‐doping increase DPPH scavenging activity. ABTS^•+^ can stable RNS, and its solution has a characteristic absorption peak at 734 nm.^[^ ^30^ ^]^ After mixed with Fe‐VO_x_, the remarkable absorbance peak decrease could be observed, and the ABTS^•+^ scavenging performance of Fe‐VO_x_ was concentration dependent (Figure S12, Supporting Information). Fe‐VO_x_ had a better ABTS^•+^ scavenging performance compared with VO_x_ (Figure 3p). Fe‐VO_x_ had a better RNS scavenging activity than VO_x_ could be attributed to the synergistic effect of dual active sites and Fe‐doped nanozymes with higher defect concentration.

### 2.3 DFT Calculation

**Figure 4:**
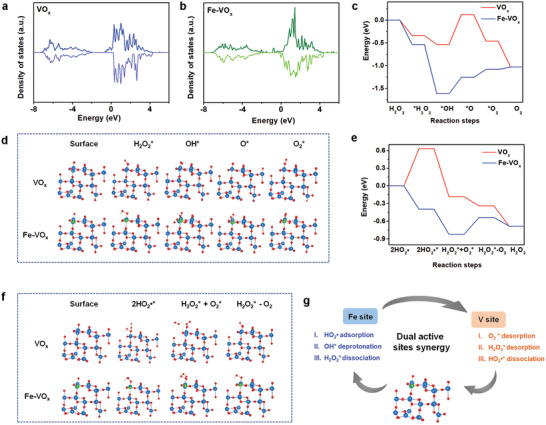
DFT calculation study on ROS‐elimination activities of Fe‐VO_x_. a) VO_x_ and b) Fe‐VO_x_. c,d) Free energy diagrams and surface structure models and of VO_x_ and Fe‐VO_x_ in the CAT‐like reaction process. e,f) Free energy diagrams and surface structure models and of VO_x_ and Fe‐VO_x_ in the SOD‐like reaction process. g) Schematic illustration of the synergistic catalysis mechanism for enzyme‐mimic catalysis on Fe‐VO_x_ nanozymes.

Based on the above results of multienzyme‐like catalyzed reactions, we used DFT calculation with the Vienna ab initio simulation package code (VASP) to elucidate the plausible CAT and SOD‐like catalytic mechanisms of Fe‐VO_x_. Projected density of states (PDOS) indicated that the d‐band center of the V orbital of Fe‐VO_x_ was −0.906 eV, which was closer to the Fermi level compared to VO_x_ (−1.087 eV) that could be ascribed to Fe doping leading to charge redistribution (**Figure** **4**a,b). The d‐band center upshift illustrated that the interaction of the adsorbed substrate with the nanozymes surface was reinforced, and then enhancing the catalytic reaction.

The reaction intermediate of CAT‐like catalysis at different stages is shown in Figure 4c,d. The first reaction process involved H_2_O_2_ adsorption and activation, and then homolysis to generate reactive OH^*^ on the active centers. OVs had strong coupling with H_2_O_2_ and acted as the preferred binding site for homolysis transformation into the OH^*^ intermediate.^[^ ^25^ ^]^ The thermodynamically energy change illustrated that the deprotonation of OH^*^ and form O^*^ process was the rate‐determining step. Fe‐induced active sites had been confirmed with mild binding affinity with OH^*^ and facilitate H^*^ abstraction.^[^ ^31^ ^]^ For the transformation of O^*^ to O_2_, the easier O─O bonding formation on VO_x_ with energy of −0.587 eV, compared with Fe sites (0.174 eV). Besides, the desorption of O_2_^*^ from VO_x_ surface to generate O_2_ with energy −0.567 eV, indicating that V sites could facilitate O_2_ desorption.

•O_2_−, a Brønsted base, has ability to bonding with a proton of H2O to form HO_2_•,^[^ ^32^ ^]^ as in Equation (1):

(1) $${•O}_{2}^{-}+H_{2}O=HO_{2}•+OH^{-}$$

Therefore, HO_2_• adsorption on the surfaces of nanozymes as model to perform SOD‐like activity catalyzed reaction simulation. The structure change of two HO_2_•^*^ is as follows:

(2) $$2HO_{2}*=O_{2}\;*+H_{2}O_{2}*$$

As shown in Figure 4e,f, the HO_2_• was more easily adsorbed on Fe‐VO_x_ with energy of −0.398 eV compared with VO_x_ (0.632 eV), indicating that Fe sites could facilitate HO_2_• adsorption. The higher energy barrier of HO_2_• → HO_2_•^*^ step limited the SOD activity of VO_x_. The O_2_ desorption from surface had small uphill energy of 0.287 eV, which is rate‐determining step of Fe‐VO_x_ induced SOD. As a result, Fe sites facilitated the initial HO_2_• adsorption, and the V sites promoted the subsequent step.

The dual sites synergy is shown in Figure 4g. In brief, Fe sites favor the adsorption of HO_2_•, deprotonation of OH^*^, and dissociation of H_2_O_2_^*^. Besides, the V sites facilitate dissociation of HO_2_•^*^ and desorption of O_2_^*^ and H_2_O_2_^*^. The DFT calculation results were echoed with the previous kinetic investigations. RNS scavenging ability of nanozymes was further investigated by DFT. As shown in Figure S13 (Supporting Information), the stronger adsorption of •NO and ONOO^−^ on Fe‐VO_x_ compared with VO_x_. The strong attraction was conducive to the dissociation of RNS.

### 2.4 Photothermal Property

**Figure 5:**
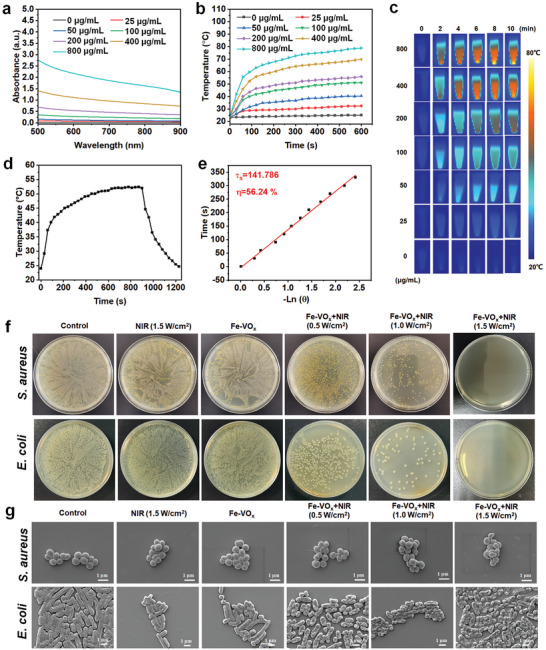
Photothermal property and antibacterial ability of Fe‐VO_x_. a) Vis−NIR absorbance spectra of Fe‐VO_x_. b) Temperature variation curve of Fe‐VO_x_ nanozymes at different concentrations after 808 nm laser 1.5 W cm^−2^) irradiation and corresponding c) photothermal images. d) Heating and cooling curve of Fe‐VO_x_. e) The linearity curves about time and ‐Ln(θ) fitted from the temperature cooling process of Fe‐VO_x_. The antibacterial effect of the different treatments on *S. aureus* and *E. coli* was evaluated by f) colony counting and g) SEM images of bacteria morphology.

The photothermal effect of Fe‐VO_x_ was first investigated by UV‐vis‐NIR spectroscopy. An obvious optical absorption ≈808 nm for Fe‐VO_x_ (**Figure** **5**a). Afterward, Fe‐VO_x_ suspensions of different concentrations were exposed with NIR laser and temperature recorded by thermal imagery. As shown in Figure 5b,c, the apparent temperature increase of Fe‐VO_x_ suspension could be found and showed concentration dependence. As shown in Figure S14 (Supporting Information), compared with VO_x_, after 10 min NIR laser irradiation with powder density of 1.5 W cm^−2^, the temperature increase of Fe‐VO_x_ is much higher than VO_x_, indicating that the defect improves photothermal performance of nanozymes. To further assess the photothermal reversibility of Fe‐VO_x_, repeated cycles of NIR laser irradiation were performed (Figure S15, Supporting Information). Almost no significant decline of photothermal performance could be observed during the five cycles. The photothermal conversion efficiency (𝜂) of Fe‐VO_x_ was 56.24% according to the linear time data (Figure 5d,e). In vivo infrared thermal images of Fe‐VO_x_+NIR group illustrated that the temperature of the jawbone could reach to 52.6 °C after exposed with NIR laser for 10 min (Figure S16, Supporting Information), which further indicated nanozymes with good photothermal conversion capacity.

### 2.5 In Vitro Antibacterial Effect of Fe‐VO_x_

To evaluate photothermal antibacterial ability of Fe‐VO_x_ under NIR laser, colony‐forming unit (CFU) of *Staphylococcus aureus* (*S. aureus*) and *Escherichia coli* (*E. coli*) were performed. As shown in Figure 5f, almost no remarkable change of CFU between the control, NIR (1.5 W cm^−2^), and Fe‐VO_x_ groups, indicating that the pure NIR laser or nanozymes without antibacterial activity. After Fe‐VO_x_ exposed with NIR laser, the numbers of colonies were significantly reduced with irradiation power increase. The photothermal antibacterial activity of Fe‐VO_x_ was in a concentration‐dependent (Figure S17, Supporting Information). SEM images of bacterial morphology also revealed similar result (Figure 5g). For Fe‐VO_x_ + NIR group, bacterial morphology appeared obvious changes. The most of bacteria were irregular or cracked in Fe‐VO_x_ +NIR (1.5 W cm^−2^) group. In contrast, because the poor photothermal conversion ability of VO_x_, almost no antibacterial effect of VO_x_+NIR could be found (Figure S18, Supporting Information). In addition, methicillin‐resisting *Staphylococcus aureus* (*MRSA*) was further used to assess the photothermal antibacterial effect of Fe‐VO_x_. As shown in Figure S19 (Supporting Information), almost no colonies of *MRSA* could be observed in Fe‐VO_x_+NIR (1.5 W cm^−2^) group indicating that *MRSA* could be effectively inhibited via photothermal effect.

### 2.6 Biocompatibility and Hyperproliferation of Fe‐VO_x_

**Figure 6:**
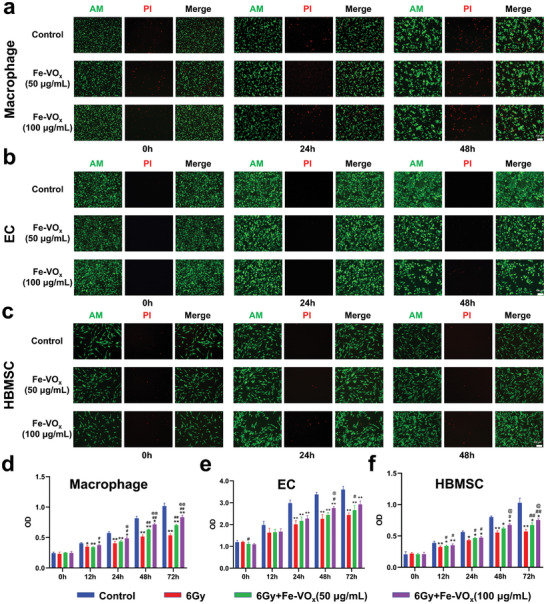
In vitro biosafety and hyperproliferation of Fe‐VO_x_. Live/Dead dual‐stained fluorescence images of a) macrophage, b) EC and c) HBMSC treated with different concentrations of Fe‐VO_x_ at 0, 24, and 48 h, scale bars, 100 µm (*n* = 3). Cell viabilities of d) macrophage, e) EC and f) HBMSC in the presence of 6 Gy radiation and different concentrations of Fe‐VO_x_, ^**^ *p* < 0.01 and ^*^ *p* < 0.05 versus the control group, ^##^ *p* < 0.01 and ^#^ *p* < 0.05 versus the 6 Gy group, ^@@^ *p* < 0.01 and ^@^ *p* < 0.05 versus the 6 Gy +Fe‐VO_x_ (50 µg mL^−1^) group (*n* = 3).

The Jawbone microenvironment contains multiple and heterogeneous cellular component. We recently proposed the concept of a jaw vascular unit (JVU) and emphasized the importance of the JVU components in inflammatory diseases of the jawbone.^[^ ^33^ ^]^ Macrophage, EC, and HBMSC are significant in the JVU, and are closely linked to ORNJ. The accumulation of RONS induced by radiation disrupts the normal polarization of macrophage and enhances proinflammatory properties.^[^ ^34^ ^]^ Hypovascular caused by radiation reduces new blood vessels forming, which is essential for nourishing jawbone. Radiation also diminishes osteogenic differentiation of HBMSCs and aggravates jawbone destruction.^[^ ^4^ ^]^ To further evaluate and guarantee the impact of Fe‐VO_x_, different concentrations of Fe‐VO_x_ were incubated with the above three types of cells. The cellular biocompatibility of Fe‐VO_x_ was examined, and cellular calcein‐AM/propidium iodide (PI) staining suggested that the number of PI‐accessible cells (cells with damaged membranes) was not significantly reduced in Fe‐VO_x_ groups (**Figure** **6**a–c). To further determinate the influence of radiation, macrophage was treated with different radiation doses and ascertained using a cell counting kit‐8 (CCK‐8) assay. It is found that the macrophage cell viability decreases with the increasing radiation doses, especially at 8 and 10 Gy (Figure S20, Supporting Information). Thus, three in vitro models of cell injury were established by irradiating macrophage, EC and HBMSC with a single dose of 6 Gy, as previously reported.^[^ ^35^ ^]^ Hyperproliferation of three cells by Fe‐VO_x_ was detected by CCK‐8 assay and can be clearly observed (Figure 6d–f). Significant decrease in cell viability was detected in three cells after radiation. Nevertheless, increase in cell viability was observed after the addition of Fe‐VO_x_, and the cell proliferation upregulated with the elevated Fe‐VO_x_ concentration, thereby indicating that the Fe‐VO_x_ could efficiently reverse cell death caused by radiation.

### 2.7 In Vitro RONS Scavenging and Cellular Protecting

**Figure 7:**
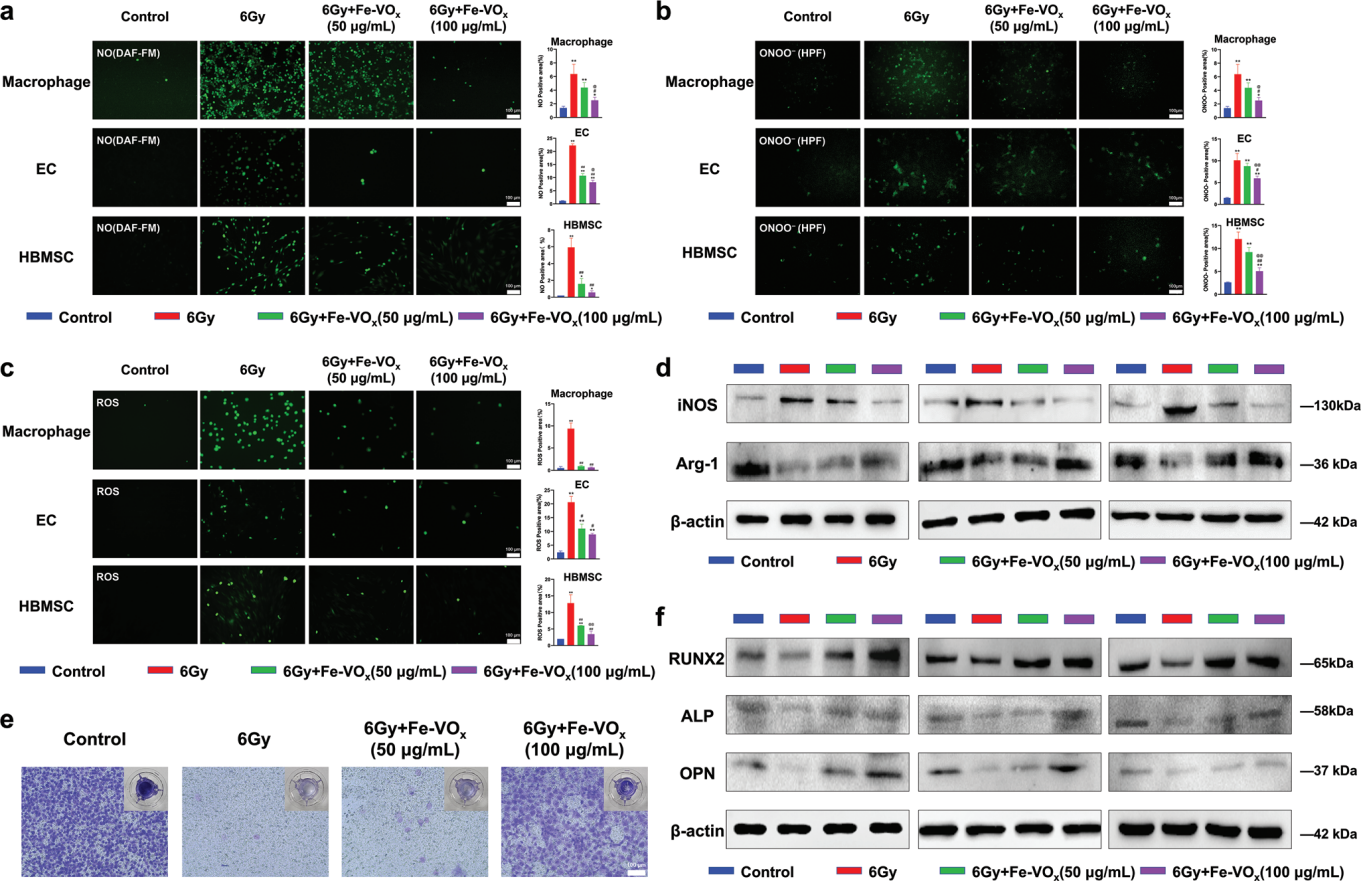
RONS scavenging activity of Fe‐VO_x_ protects cellular function against radiation injury. Fluorescence micrographs and intensity quantification showing the activities of Fe‐VO_x_ to scavenge different types of RONS, including a) NO, b) ONOO^−^, and c) ROS in macrophage, EC and HBMSC with 6 Gy radiation pretreatment, scale bars, 100 µm, ^**^ *p* < 0.01 and ^*^ *p* < 0.05 versus the control group, ^##^ *p* < 0.01 and ^#^ *p* < 0.05 versus the 6 Gy group, ^@@^ *p* < 0.01 and ^@^ *p* < 0.05 versus the 6 Gy+Fe‐VO_x_ (50 µg mL^−1^) group. (*n* = 3). d) Protein expressions of iNOS and Arg‐1 in macrophage were determined by western blot analysis (*n* = 3). e) Representative images of Transwell migration assays of EC, scale bars, 100 µm. f) Protein expressions of RUNX2, ALP and OPN in HBMSC were determined by western blot analysis (*n* = 3).

As a radiation‐sensitive organ, the jaw bone is susceptible to radiation‐induced sepsis exposure, leading to stomatognathic system dysfunction.^[^ ^36^ ^]^ RONS production is closely related to sepsis‐related organ damage.^[^ ^37^ ^]^ Inspired by multienzyme‐like activities of Fe‐VO_x_, we carefully explored its therapeutic potential in scavenging RONS and recovering physiological functions in radiation‐induced microenvironment. The effect of Fe‐VO_x_ on cellular RNS levels was first detected by 3‐amino, 4‐aminomethyl‐2′,7′‐difluorescein diacetate (DAF‐FM), and hydroxyphenyl fluorescein (HPF) staining, which was knowledge‐based on that NO and ONOO^−^ are representative RNS (**Figure** **7**a,b). NO and ONOO^−^ contribute to various redox related diseases due to their nitrosative damage to lipids, proteins, and DNA.^[^ ^38^ ^]^ The fluorescence microscopy results illustrated that all three cells generated an excessive amount of RNS, after 6 Gy radiation. However, the fluorescence markedly weakened after Fe‐VO_x_ incubation, and Fe‐VO_x_ (100 µg mL^−1^) could reduce the fluorescence to almost background level, suggesting the general RNS scavenging capability. Next, cellular oxidative stress was measured. The intracellular ROS level was detected by 2′,7′‐dichlorodihydrofluorescein diacetate (DCFH‐DA) using fluorescence microscope, which was based on the knowledge that ROS can oxidize nonfluorescent DCFH to generate fluorescent DCF (Figure 7c). SOD, which could scavenge •O_2_^−^ and resist oxidative stress injuries,^[^ ^39^ ^]^ was detected by a xanthine oxidase assay kit (Figure S21, Supporting Information). Both results demonstrated an obvious oxidative stress elimination effect, which was highly consistent with the RNS observation. Collectively, the Fe‐VO_x_ exhibited the ability to scavenge various types of RONS, which could be applied as an efficient antioxidant to protect the cells from radiation‐induced oxidative damage.

Above results have firmly investigated the intracellular RONS scavenging performances of Fe‐VO_x_. In addition to directly cause organ damage, RONS could also disorganize normal tissue via inducing proinflammatory cytokines.^[^ ^40^ ^]^ We next examined the capabilities of Fe‐VO_x_ in cellular functions. Three types of cells, including macrophage, EC, and HBMSC cells were stimulated by 6 Gy radiation and treated with Fe‐VO_x_. Macrophage is important component of the immune system and susceptible to the inflammation in local microenvironment.^[^ ^41^ ^]^ Accumulated RONS contributes to oxidative destruction and reprogram macrophage polarization phenotype, which is characterized by proinflammatory properties that may abolish jawbone regeneration.^[^ ^42^ ^]^ M2 macrophage marker (ARG‐1) and M1 macrophage marker (iNOS) were detected in the presence of radiation and Fe‐VO_x_. Western blot analysis found that iNOS levels were upregulated by radiation, but downregulated by Fe‐VO_x_, especially in Fe‐VO_x_ (100 µg mL^−1^) groups. In contrast, Arg‐1 expression exhibited an opposite trend (Figure 7d; Figure S22, Supporting Information). Subsequently, reversal of radiation injury by Fe‐VO_x_ was assessed in EC and HBMSC, respectively. EC facilitates the blood vessels forming and secretes valuable angiogenesis factors to nourish jawbone.^[^ ^43^ ^]^ Abnormal sepsis accumulation due to radiation would cause endothelial dysfunction via disrupted EC angiogenesis capability.^[^ ^44^ ^]^ Transwell migration and wound scratch assays showed that EC migration was decreased by radiation, while Fe‐VO_x_ significantly increased cell migration, especially in Fe‐VO_x_ (100 µg mL^−1^) groups (Figure 7e; Figure S23, Supporting Information). HBMSC, with an unlimited or immortal self‐renewal capacity, could be recruited from bone marrow to jawbone defected area and participate in new bone mineralization process.^[^ ^45^ ^]^ Previous studies revealed that radiation resulted in osteogenic differentiation disorder of HBMSC and contributed to the incomplete jawbone maturation.^[^ ^46^ ^]^ So, levels of osteogenesis‐related proteins were performed in HBMSC. Runt‐related transcription factor 2 (RUNX2) is regarded as the key osteogenic transcriptional factor.^[^ ^47^ ^]^ Alkaline phosphatase (ALP) is a key marker of early bone formation.^[^ ^48^ ^]^ Osteopontin (OPN) directly enhancing the process of osteanagenesis.^[^ ^49^ ^]^ Western blot analysis showed that these three proteins were inhibited by radiation, while Fe‐VO_x_ significantly promoted their expression, especially in Fe‐VO_x_ (100 µg mL^−1^) groups (Figure 7f; Figure S24a–c, Supporting Information). Taken together, Fe‐VO_x_ effectively protected cellular function dysfunction by switching macrophage from M1 to M2 phenotype, eliminating angiogenesis dysfunction and reversing osteogenesis disorder. We further detected the valuable cytokines excreted by macrophage by Enzyme‐linked immunosorbent assay (ELISA). IL‐1β is regarded as a proinflammatory marker and TGF‐β1 is regarded as an anti‐inflammatory marker. Our results showed that Fe‐VO_x_ significantly inhibited IL‐1β and promoted TGF‐β1 expressions in radiation‐treated macropgahe (Figure S24d, Supporting Information), which indicated the ability of RONS scavenging to disorganize normal tissue via proinflammatory and anti‐inflammatory cytokines. In addition, protein expressions of BCL2 associated X (BAX), Gasdermin‐D (GSDMD), and Tumor necrosis factor‐alpha (TNF‐α) in macrophage were measured by western blot analysis and the results suggested that Fe‐VO_x_ might repair cellular dysfunction via apoptosis and pyroptosis‐related pathways (Figure S24e,f, Supporting Information). The underlying mechanism needs to be investigated in further study.

### 2.8 Therapeutic Mechanisms of Fe‐VO_x_ on Radiation Injury

**Figure 8:**
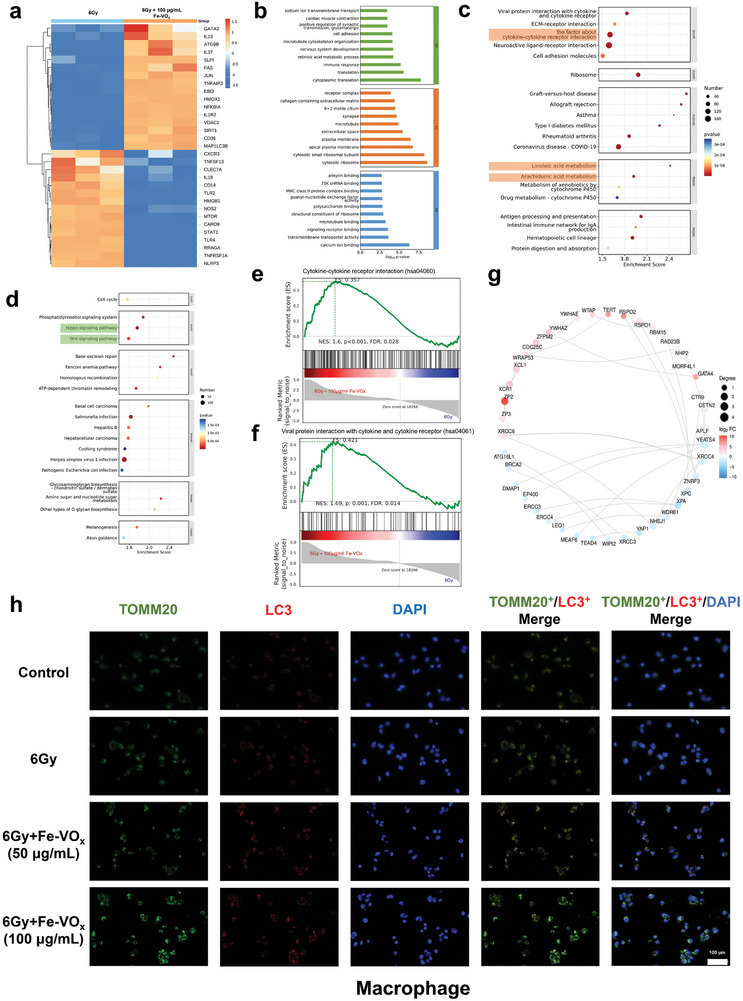
Transcriptomics analyses and mitophagy determination. a) Heatmap illustrating the expression of genes related to inflammation, autophagy, and mitophagy in 6Gy‐induced macrophage with or without Fe‐VO_x_ (100 µg mL^−1^) treatment. b) GO analysis highlighting the biological processes affected by differentially expressed genes. KEGG pathway analysis of upregulated c) and downregulated d) genes in the Fe‐VO_x_ treatment group. GSEA results for cytokine‐cytokine receptor interaction e) and viral protein interaction with cytokine and cytokine receptor f) pathways. g) PPI network analysis displaying interactions among proteins encoded by differentially expressed genes in macrophage with Fe‐VO_x_ treatment. IF staining showing the TOMM20^+^/ LC3^+^ colocalization in (h) macrophage with or without Fe‐VO_x_ (50 or 100 µg mL^−1^) treatment after 6 Gy radiation, scale bars, 100 µm (*n* = 3).

The above results demonstrated an obvious therapeutic effect with regard to RONS scavenging and cellular dysfunction restoring. We further utilized transcriptomics analyses of 6Gy‐induced macrophage with or without Fe‐VO_x_ (100 µg mL^−1^) treatment to comprehensively reveal the protective process and understand the underlying regulatory mechanism of Fe‐VO_x_ in treating ORNJ. First, we performed quality control on the sequencing results, which indicated that the data met the required standards (Figure S25a, Supporting Information). Differential gene expression analysis revealed that, in macrophage treated with Fe‐VO_x_, 10 874 genes were upregulated, while 4073 genes were downregulated (Figure S25b, Supporting Information). Using the criteria of *p* < 0.05 and |log2FC|>1, 7990 genes were found to be upregulated, and 6152 genes were downregulated (Figure S25c, Supporting Information). Further analysis of these differentially expressed genes (DEGs) revealed that pro‐inflammatory genes such as *IL18* and *TNFSF13* ^[^ ^50^ ^]^ were downregulated, while anti‐inflammatory genes like *IL13* ^[^ ^51^ ^]^ were upregulated, suggesting that Fe‐VO_x_ effectively suppressed inflammation induced by radiation. Additionally, autophagy‐related genes *ATG9B* and *MAP1LC3B* ^[^ ^52^ ^]^ were significantly upregulated, along with the mitophagy‐related gene *SIRT1*,^[^ ^53^ ^]^ indicating an enhancement in both autophagy and mitophagy levels in macrophage following Fe‐VO_x_ treatment (**Figure** **8**a).

Next, we conducted functional analysis of the DEGs. Gene Ontology (GO) analysis revealed obvious differences in several biological processes (Figure 8b; Figure S26a,b, Supporting Information). Kyoto encyclopedia of genes and genomes (KEGG) analysis indicated that, compared to the 6 Gy groups, cytokine‐cytokine receptor interaction was upregulated in Fe‐VO_x_‐treated groups, which has a dual role in regulating inflammation^[^ ^54^ ^]^ (Figure S26c, Supporting Information). Additionally, the metabolism of linoleic acid and arachidonic acid was also upregulated. Linoleic acid is known to promote autophagy,^[^ ^55^ ^]^ while arachidonic acid and its derivatives can enhance both autophagy and mitophagy^[^ ^56^ ^]^ (Figure 8c). Conversely, the Wnt and Hippo pathways, both of which are inflammation‐related,^[^ ^57^ ^]^ were downregulated in Fe‐VO_x_‐treated groups (Figure 8d). Gene set enrichment analysis (GSEA) analysis further revealed that cytokine‐cytokine receptor interaction (Figure 8e) and viral protein interaction with cytokine and cytokine receptor (Figure 8f) were both upregulated along with Fe‐VO_x_ treatment, consistent with the KEGG analysis results. Besides, protein‐protein interaction networks (PPI) analysis identified interactions among proteins encoded by anti‐inflammatory genes (*XCR1* and *ZP2*)^[^ ^58^ ^]^ and pro‐inflammatory genes (*BRCA2*, *XPC*, and *ERCC4*).^[^ ^59^ ^]^ *BRCA2* was also reported to promote mitophagy through the Fanconi anemia pathway (Figure 8g).^[^ ^60^ ^]^ Collectively, these finding illustrated the potential capability of Fe‐VO_x_ in reprogramming inflammatory microenvironment and mitophagy against radiation injury.

Inspired by the transcriptional profiles, GSEA, and PPI results, we assumed that RONS scavenging‐enhanced mitophagy might be the key mechanism of the Fe‐VO_x_ therapeutic effects. Oxidative damage caused by radiation contributes to dysfunctional or excessive mitochondria, which could be selective degraded, and cleaned up by mitophagy.^[^ ^61^ ^]^ TOMM20 is a classic outer mitochondrial membrane protein and HSP60 is regarded as mitochondrial chaperones, while LC3 is often used as a marker for autophagosomes.^[^ ^62^ ^]^ Given the involvement of above proteins in the mitophagy process, we subsequently investigate their expression in 6Gy‐induced cells with or without Fe‐VO_x_. Immunofluorescence (IF) staining revealed that the 6 Gy radiation induced massive mitophagy suppression, while treatment with Fe‐VO_x_ (especially 100 µg mL^−1^) significantly promoted the mitophagy level, which is manifested as the TOMM20^+^/ LC3^+^ colocalization, all in macrophage, EC and HBMSC (Figure 8h; Figure S27, Supporting Information). Simultaneously, the HSP60^+^/ LC3^+^ colocalization inhibited by 6 Gy radiation was remarkably alleviated by the administration of Fe‐VO_x_ (especially 100 µg mL^−1^) (Figures S28–S30, Supporting Information). Overall, these results indicated that Fe‐VO_x_ effectively upregulated mitophagy through restoring mitochondrial dysfunction and exerting valuable protective effects during radiation injury.

### 2.9 In Vivo Therapeutic Assessment of Fe‐VO_x_

**Figure 9:**
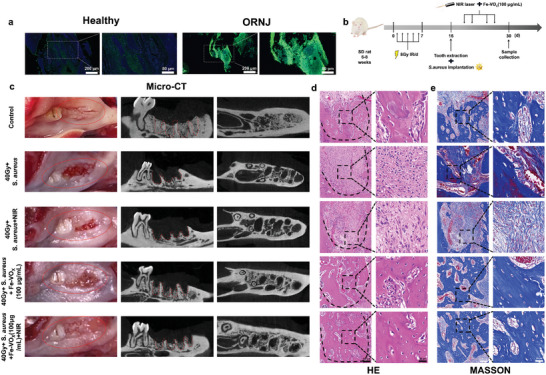
Radioprotection of Fe‐VO_x_ alleviates ORNJ. a) Representative images of ROS staining of mandible sections from both healthy individual and ORNJ patient, scale bars, 200 or 80 µm. The experiment was repeated three times independently with similar results. b) Schematic illustration for in vivo ORNJ model establishing and radioprotection evaluation. c) Clinical appearance of the gingival mucosa and bone formation in TESs. Red solid lines outline the gingival mucosa. Red dotted lines outline the extraction sockets. (*n* = 3). d) HE staining of bone trabecula (black box) in TESs (black dotted line), scale bars, 100 or 25 µm (*n* = 3). e) Masson's staining of collagenous fibers (black box) in TESs, scale bars, 100 or 25 µm (*n* = 3).

Encouraged by the favorable RONS scavenging and radioprotective properties of Fe‐VO_x_, in vivo ORNJ therapy was further assessed. Mandible samples from both healthy individuals and ORNJ patients were first analyzed to investigate the correlation between oxidative stress and ORNJ. IF staining revealed that compared with healthy group, tissue ROS level was significantly upregulated in ORNJ group (**Figure** **9**a). Data from the hematoxylin & eosin (HE) and Masson's staining further showed reduced bone trabecula and collagen deposition in the ORNJ group than in the healthy group (Figures S31a,b and S32a,b, Supporting Information). These results indicate a close relationship between oxidative stress and the severity of ORNJ. Next, we established an ORNJ model of Sprague–Dawley (SD) rat with NIR laser and Fe‐VO_x_ (100 µg mL^−1^) local injection for 14 days after 7 days of radiation, 10 days of rest, extraction of the first and second molars of the mandible and *S. aureus* topically implanting (Figure 9b). Microcomputed tomography (micro‐CT) analysis was used to 3D reconstruct alveolar bone formation in the TESs on day 14 after teeth extraction. Decreased alveolar bone formation was observed in the 40Gy+*S. aureus* and 40 Gy+*S. aureus*+NIR groups compared to that in the control group. Intriguingly, the TESs of 40Gy+*S. aureus*+Fe‐VO_x_ (100 µg mL^−1^) or 40Gy+*S. aureus*+Fe‐VO_x_ (100 µg mL^−1^) +NIR treated rats exhibit preferable bone formation compared with the 40Gy+*S. aureus* and 40Gy+*S. aureus*+NIR groups. In addition, 40Gy+*S. aureus*+Fe‐VO_x_ (100 µg mL^−1^) +NIR group showed favorable mucosa healing compared with other groups, which is critical for preventing oral cavity pathogens into TES and making room for new bone growth^[^ ^63^ ^]^ (Figure 9c; Figure S31c, Supporting Information). Furthermore, HE and Masson's staining showed suppressed bone trabecula and collagen deposition induced by radiation and *S. aureus* were reversed by Fe‐VO_x_(100 µg mL^−1^) and NIR (Figure 9d,e; Figure S32c, Supporting Information). To investigate in vivo antibacterial effects, fluid from jawbone of different groups were used for bacteria count. As shown in Figure S33 (Supporting Information), neither of pure NIR exposure nor Fe‐VO_x_ have antibacterial ability. In contrast, very few bacterial colonies could be found in Fe‐VO_x_+NIR group, which indicated that Fe‐VO_x_ with excellent in vivo antibacterial activity. Considering the biosafety and toxicity are the premise of clinical application, HE staining of the major organ tissues (heart, liver, spleen, lung, and kidney) was carried out and the results showed no noticeable abnormalities among each group (Figure S34, Supporting Information). To explore in vivo distribution of Fe‐VO_x_ nanozymes. As shown in Figure S35 (Supporting Information), V element were mainly in liver, kidney, and spleen via ICP‐MS measurement and V content was decreasing over time indicated that Fe‐VO_x_ could be smoothly excreted. Overall, bone deficiency‐inhibiting property and outstanding biocompatibility identified Fe‐VO_x_ as an effective and sage therapeutic biomaterial for preventing ORNJ.

**Figure 10:**
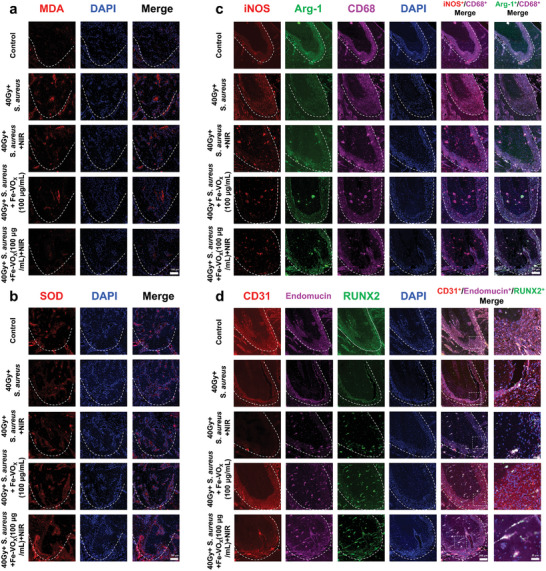
Fe‐VO_x_ protects against radiation‐impaired jawbone microenvironment by attenuating oxidative stress. Representative images of IF staining of a) MDA and b) SOD in TESs (white dotted line), scale bars, 100 µm (*n* = 3). c) Representative images of IF staining of iNOS, Arg‐1 and CD68 in TESs (white dotted line), scale bars, 100 µm (*n* = 3). d) Representative images of IF staining of CD31, Endomucin and RUNX2 in TESs (white dotted line), scale bars, 100 or 25 µm (*n* = 3).

Since ROS was significantly upregulated in ORNJ clinical samples, we subsequently reconfirmed the antioxidation of Fe‐VO_x_ in vivo. It is known that lipid peroxidation malondialdehyde (MDA) and SOD activity are representative of the oxidative stress level. MDA reflects the lipid peroxidation level induced by ROS, while SOD could scavenge elevated superoxide species and resist oxidative stress injuries, further protect impaired tissue.^[^ ^39^ ^]^ Based on this, IF staining was conducted to detect MDA and SOD levels in animal models. Increased MDA and decreased SOD levels were observed in the 40Gy+*S. aureus* and 40Gy+*S. aureus*+NIR groups compared to that in the control group. Conversely, downregulated MDA and upregulated SOD were found in 40Gy+*S. aureus*+Fe‐VO_x_ (100 µg mL^−1^) or 40 Gy+*S. aureus*+Fe‐VO_x_ (100 µg mL^−1^) +NIR groups, compared with the 40Gy+*S. aureus* and 40Gy+*S. aureus*+NIR groups (**Figure** **10**a,b; Figure S36a,b, Supporting Information). This result suggests that the positive effects of Fe‐VO_x_ may be due to the scavenging of oxidative stress, alleviating bone deficiency.

To address the therapeutic effects of Fe‐VO_x_ on radiation‐impaired jawbone microenvironment, IF staining was utilized to detect the biomarkers of macrophage polarization, angiogenesis, and osteogenesis. It is well known that M1 macrophage aggravate alveolar bone loss via secreting pro‐inflammatory cytokines, whereas M2 macrophage attenuate bone loss through anti‐inflammatory properties.^[^ ^64^ ^]^ Herein, we found that increased M1 macrophage (iNOS^+^/CD68^+^ colocalization) and decreased M2 macrophage (Arg‐1^+^/CD68^+^ colocalization) were observed in the 40Gy+*S. aureus* group compared to that in the control group. In contrast, downregulated M1 macrophage and upregulated M2 macrophage were found in 40Gy+*S. aureus*+Fe‐VO_x_ (100 µg mL^−1^) or 40Gy+*S. aureus*+Fe‐VO_x_ (100 µg mL^−1^) +NIR groups, compared with the 40Gy+*S. aureus* and 40Gy+*S. aureus*+NIR groups (Figure 10c; Figure S36c,d, Supporting Information). Besides, evidence by CD31^+^/ Endomucin^+^ colocalization suggested radiation‐impaired angiogenesis was reversed by Fe‐VO_x_, which was based on the knowledge that CD31 and Endomucin were classical markers of type H vessel, a unique type of blood vessel intimately coupled with alveolar bone formation.^[^ ^65^ ^]^ Additionally, as the primary regulatory factor of osteogenic differentiation of BMSC and recruiter of type H vessel,^[^ ^66^ ^]^ reduced RUNX2 expression in 40Gy+*S. aureus* group was also reversed by Fe‐VO_x_ administration (Figure 10d; Figure S36e, Supporting Information). Moreover, TOMM20^+^/ LC3^+^ colocalization in TESs indicated mitophagy inhibited by radiation was remarkably alleviated by the administration of Fe‐VO_x_ (Figure S37, Supporting Information). These above results highlighted the immune‐angiogenesis‐osteogenesis synergism of Fe‐VO_x_ in alleviating radiation‐impaired jawbone microenvironment, further confirmed the in vitro analysis at the cellular level.

## 3 Conclusion

In summary, we developed a novel antioxidative nanozyme with CAT, SOD, and GPx‐like three enzymatic activities, Fe‐VO_x_, for ORNJ therapy. The excellent enzyme‐mimicking catalytic activities of Fe‐VO_x_ nanozymes were due to dual active sites synergy and defect engineering. DFT calculations disclosed that Fe sites contribute the adsorption of HO_2_•, deprotonation of OH^*^ and dissociation of H_2_O_2_^*^. And V sites facilitates dissociation of HO_2_•^*^ and desorption of O_2_^*^ and H_2_O_2_^*^. The theoretical calculation results were in line with Michaelis–Menten kinetics. In vitro and in vivo experiments have shown that Fe‐VO_x_ with good biocompatibility could eliminate RONS of jawbone microenvironment. The properties of mitophagy activation and photothermal antibacterial which make Fe‐VO_x_ nanozymes have a clinical application promising.

## Conflict of Interest

The authors declare no conflict of interest.

## Supporting information



Supporting Information

## Data Availability

The data that support the findings of this study are available from the corresponding author upon reasonable request.
