# Supplementary material for: Engineering Dual Active Sites and Defect Structure in Nanozymes to Reprogram Jawbone Microenvironment for Osteoradionecrosis Therapy
Source: Adv Sci (Weinh). 2024 Dec 17;12(6):2413215. doi: 10.1002/advs.202413215 (PMC11809426; doi:10.1002/advs.202413215)
Supplement: Supplementary file 1 — Supporting Information [file ADVS-12-2413215-s001.docx]

**Supporting Information**

**Engineering Dual Active Sites and Defect Structure in Nanozymes to** **Reprogram Jawbone Microenvironment for Osteoradionecrosis Therapy**

*Zheng Cheng^1^, Yuchen Wang^1^, Haobo Lin^1^,* *Ziyu Chen^2^, Ran Qin^1^, Tianxiao Wang^2^,* *Hang Xu^3^, Yifei Du^1^, Hua Yuan^1^, Yongchu Pan^4^, Huijun Jiang^2^, Xinquan Jiang^5^, Jiandong Jiang^2,6^, Fan Wu^2*^, Yuli Wang^1,2*^*

^1^Department of Oral and Maxillofacial Surgery, The Affiliated Stomatological Hospital of Nanjing Medical University; State Key Laboratory Cultivation Base of Research, Prevention and Treatment for Oral Diseases; Jiangsu Province Engineering Research Centre of Stomatological Translational Medicine, Nanjing Medical University, Nanjing Jiangsu 210029, China

^2^Medical Basic Research Innovation Centre for Cardiovascular and Cerebrovascular Diseases, Ministry of Education; International Joint Laboratory for Drug Target of Critical Illnesses; School of Pharmacy, Nanjing Medical University, Nanjing Jiangsu 211166, China

^3^State Key Laboratory of Systems Medicine for Cancer, Shanghai Cancer Institute, Renji Hospital Affiliated to Shanghai Jiao Tong University School of Medicine, Shanghai 200120, China.

^4^Department of Orthodontic, The Affiliated Stomatological Hospital of Nanjing Medical University; State Key Laboratory Cultivation Base of Research, Prevention and Treatment for Oral Diseases; Jiangsu Province Engineering Research Centre of Stomatological Translational Medicine, Nanjing Medical University, Nanjing Jiangsu 210029, China

^5^Department of Prosthodontics, Shanghai Ninth People's Hospital, Shanghai Jiao Tong University School of Medicine, College of Stomatology, Shanghai Jiao Tong University, Shanghai Engineering Research Center of Advanced Dental Technology and Materials, National Center for Stomatology, National Clinical Research Center for Oral Diseases, Shanghai Key Laboratory of Stomatology, Shanghai Research Institute of Stomatology, No. 639 Zhizaoju Road, Shanghai, 200011, China

^6^Institute of Medicinal Biotechnology; Chinese Academy of Medical Sciences & Peking Union Medical College, Beijing 100050. China

**Corresponding Author:**

Fan Wu, Email: [wufan71@njmu.edu.cn](mailto:wufan71@njmu.edu.cn)

Yuli Wang, Email: [njykdwyl@njmu.edu.cn](mailto:njykdwyl@njmu.edu.cn)

**Materials and methods**

**Materials.** Anhydrous ferric chloride, hydrogen peroxide, hydrochloric acid, and ethanol were purchased from Sinopharm Chemical Reagent Co., Ltd. Tannic acid, ammonium metavanadate, glutathione, 3,3'-Dithiobis(6-nitrobenzoic acid), DPPH, ABTS, PTIO were purchased from Aladdin Reagent Co., Ltd.

**Characterization of nanozymes.** The visualizations of nanozymes were performed by using AFM (Bruker Dimension Icon) and TEM (JEOL, JEM-F200). DLS of samples was measured by Zetasizer Nano-ZS90 (Malvern). X-ray diffraction (XRD) patterns were obtained by using Bruker D2 Phaser. UV-vis spectra of samples were measured by using UV-vis spectrophotometer (Shimadzu UV-2600i). X-ray photoelectron spectra (XPS) were performed on a X-ray photoelectron spectrometer (Thermo Fisher Nexsa). EPR spectra were got by Bruker EMXplus spectrometer.

**Synthesis of Fe-VO_x_.** Ferric chloride (0.05 g), tannic acid (0.15 g) were fully dissolved in 15 mL of deionized water by magnetic stirring at room temperature. Meanwhile, dissolving the ammonium metavanadate (0.135 g) in 15 mL of deionized water by magnetic stirring at 60 ℃. Then, both solution was thoroughly mixed in a 60 ℃ water bath, followed by adding hydrochloric acid (40 μL). Finally, the obtained mixture was transferred to a Teflon-lined autoclave. The mixture was heated to 200 °C for 12 h. Once cooled to room temperature, the solution was centrifuged at 12000 rpm for 15min. The precipitates were washed three times with ultrapure water and ethanol. At last, Fe-VO_x_ were obtained after vacuum drying. Compared with Fe-VO_x_, VO_x_ synthesis does not add ferric chloride and other steps remain unchanged.

**CAT-like activity of Fe-VO_x_.** A dissolved oxygen meter (JPB-607A+, Yuwo, Shanghai, China) was used to measure the cumulative release of O_2_, after nanozymes mixed with H_2_O_2_ at room temperature.

**SOD-like activity of Fe-VO_x_.** The ability of nanozymes quenching •O_2_^-^ was investigated by measuring the inhibition of formazan formation through WST-1 using a colorimetric SOD assay kit according to the manufacturer’s instructions *(A001-3-2, Nanjing Jiancheng Bioengineering institute)*. In brief, •O_2_^-^ was produced *via* the oxidation of xanthine with XOD. The amount of formazan generated is directly correlated to the number of •O_2_^-^ produced in the reaction system. Therefore, to verify the •O_2_^-^-scavenging ability of Fe-VO_x_ or VO_x_ with different concentrations, WST-1, xanthine and XOD were mixed, then the absorption changes of water-soluble formazan were spectrophotometrically monitored at 450 nm using UV-vis spectroscopy.

**GPX-like activity of Fe-VO_x_ and VO_x_.** To assess GPx-like activity, nanozymes with different concentration were added into GSH solution (2.7 mg/mL), followed by adding DTNB (0.08 mg/mL) and H_2_O_2_ (6 mM). After the mixture was mixed for 5 min, the absorbance at 412 nm was measured by UV-vis spectrophotometer.

**DPPH scavenging assay.** DPPH (0.07 mg/mL) solution in ethanol was prepared in advance. And then 0.75 mL of Fe-VO_x_ or VO_x_ nanozymes dispersion at different concentrations were mixed with 0.75 mL of DPPH solution for 30 min. Finally, the absorbance at 519 nm was measured *via* UV-vis spectroscopy.

**ABTS^•+^ scavenging assay.** ABTS solution (4.06 mg/mL, 1 mL) and potassium persulfate solution (0.7 mg/mL, 1 mL) were mixed in the dark for 24 h to generate ABTS^•+^. Then, the solution was diluted 50 times with PBS buffer for further reaction with Fe-VO_x_ and VO_x_. Afterward, 0.2 mL of Fe-VO_x_ or VO_x_ nanozymes dispersions at various concentrations and 0.8 mL of ABTS^•+^ solution were mixed for 10 min before measurement by UV−vis spectroscopy at 734 nm.

**PTIO assay.** approximately 0.05 mg/mL of PTIO solution was prepared in PBS buffer. 0.75 mL of nanozymes dispersions at various concentrations (12.5-200 μg/mL) were mixed with 0.75 mL of PTIO solution (0.05 mg/mL) and incubated at room temperature for 120 min. The absorbance of PTIO at 557 nm was measured *via* UV-vis spectroscopy.

**Photothermal effect of Fe-VO_x_.** Fe-VO_x_ suspension of different concentration were treated with 808 nm NIR laser (1.5 W/cm^2^) for 10 min to measure the photothermal effect. In the progress, we used an IR thermal camera to record the changes of temperature. The calculation of photothermal conversion efficiency was based on the the following equations:

$\eta=\frac{hS(T_{max}-T_{max, H_{2}O})}{I(1-10^{-A_{808}})}$ (3)

$hS=-\frac{\sum_{i} m_{i}C_{i}}{\tau_{s}}$ (4)

$t=-\tau_{s}\ln\theta$ (5)

$\theta=\frac{T-T_{surr}}{(T_{max}-T_{suur})}$ (6)

***In vitro* antibacterial effect of Fe-VO_x_.** *E. coli*, *S. aureus* and *MRSA* were placed in appropriate amount of liquid culture medium and cultivated in constant temperature incubator at 37°C for 12 h. After activation, the activated bacterial suspension was subjected with different treatments. For each group, they were co-incubated at 37°C for 0.5 hours. The concentration of Fe-VO_x_ was 100 μg/mL. For PTT group, the samples were irradiated with NIR laser for 10 minutes and then co-incubating for 0.5 hours. After co-incubation, take samples with a pipette gun and transfer them onto the surface of solid culture medium. Use a spreader stick to spread the samples evenly. Place the culture plates upside down in a constant temperature incubator and incubate at 37°C for 18 hours. For the observation of SEM, bacterial subjected to various treatments were washed with PBS buffer three times. The bacteria were immediately fixed with 2.5% glutaraldehyde solution at 4 °C 4 h. The fixed bacteria were further dehydrated with a series of ethanol solutions (25%, 50%, 75%, 90%, and 100%), and then observed by SEM (JEOL, JSM-7900).

**Ethics approval.** For HBMSC collection and clinical relevance evaluation, human mandible samples from both healthy individuals and ORNJ patients were included with the approval of the Ethical Committee of the Affiliated Hospital of Stomatology, Nanjing Medical University (Approval NO. PJ2024−112−001). Written informed consent was obtained from all participants. All animal studies (including the euthanasia procedure) were performed in compliance with the regulations and guidelines of institutional animal care for Nanjing Medical University and conducted according to the IACUC guidelines (Approval NO. IACUC−2407107).

**Cell culture.** The THP-1 cell line (TIB-202) was acquired from the American Type Culture Collection (ATCC, Manassas, VA, USA) and cultured with RPMI-1640 (HyClone, Provo, UT) supplemented with 1% penicillin/streptomycin (P/S) and 10% fetal bovine serum (FBS) (all from Sigma-Aldrich, St. Louis, MO, USA). For inducing M0 macrophage, 185 ng/mL phorbol 12-myristate 13-acetate (PMA; MedChemExpress LLC, Princeton, NJ, USA**)** was added to the cell culture for 6 h when the cell density reached 8x10^5^ cells/mL. EC was obtained from AllCells Biology Technology Company Ltd. (Shanghai, China) and cultured in endothelial basal medium (EBM, AllCells). HBMSC were collected from healthy individuals’ mandible samples as previously described^1^ and cultured in α-minimum essential medium supplemented with 1% P/S and 10% FBS. Passages 3−5 were used in this study. HBMSC osteogenic differentiation was induced using osteogenic differentiation medium containing 100 nM dexamethasone, 10 mM β-glycerophosphate and 100 nM ascorbic acid (all from Sigma-Aldrich, St. Louis, MO, USA). All cells were incubated in a humidified atmosphere containing 5% CO_2_/95% air at 37°C.

**Cell radiation and treatment.** Macrophage was radiated with a dose of 2,4,6,8 and 10 Gy using a 160 kV RS2000 irradiator (Rad Source; Rad Source Technologies, Coral Springs, FL) (dose rate = 1.265 Gy·min ^-1^). EC and HBMSC were radiated with 6 Gy as a single dose. Fe-VO_x_ (50 μg/mL or 100 μg/mL) were added immediately after radiation and cells were collected at different time points. For the control group, cells underwent the same period without radiation exposure or Fe-VO_x_ treatment.

**Calcein AM/PI double staining.** Macrophage, EC and HBMSC were seeded according to 1 × 10^4^ cells/well and co-cultured with Fe-VO_x_ (50 μg/mL) and Fe-VO_x_ (100 μg/mL) for 0, 24 and 48 h. Samples were prepared as previously described^2^ and fluorescence in each group was observed under an inverted fluorescence microscope (Leica Microsystems, Mannheim, Germany).

**CCK-8 assay.** CCK-8 assay was performed using a CCK-8 reagent (Jiancheng Bioengineering Institute, Nanjing, China) according to the instructions.

**Assessment of intracellular RONS.** DAF-FM and DCFH-DA were from Beyotime (Shanghai China). HPF was from Shanghai Maokang Biotechnology Co., Ltd. (Beijing, China). Macrophage, EC and HBMSC were seeded 5 × 10^4^ cells/well and incubated overnight. Cells were radiated with 6 Gy and then treated with or without Fe-VO_x_ (50 μg/mL or 100 μg/mL) for 48 h. Subsequently, intracellular RONS levels were measured by addition of 5 μM DAF-FM (for NO), 10 μM HPF (for ONOO^-^), or 20 μM DCFH-DA (for ROS). The fluorescence in each group was observed under an inverted fluorescence microscope (Leica Microsystems, Mannheim, Germany) and fluorescence intensity was counted.

**Assessment of cellular SOD activity.** A xanthine oxidase assay kit (Jiancheng Corp, Nanjing, China) was used to detect the SOD activity according to the manufacturer’s instructions.

**Western blot analysis.** HBMSC was incubated in osteogenic differentiation medium. After 6 Gy radiation, HBMSC was treated with or without Fe-VO_x_ (50 μg/mL or 100 μg/mL) for 3 d. M0 macrophage was radiated with 6 Gy and then treated with or without Fe-VO_x_ (50 μg/mL or 100 μg/mL) for 48 h. Protein extraction and western blot analysis were performed as previously described^3^. The antibodies used are listed in Table S1. Relative densitometry analysis of the blots was carried out using ImageJ software and quantified. Uncropped blots are shown in Supplementary Fig S38-S40.

**ELISA.** To assess the effect of Fe-VO_x_ on IL-1β and TGF-β1 excretion in macrophage, culture supernatants were collected and analyzed with Elisa Assay Kit (UpingBio®, China, SYP-H0688 and SYP-H0083) as directed by the manufacturer.

**Cell migration assay.** EC migration was measured by transwell migration and wound scratch assays. 5×10^4^ EC were suspended in 200 μL serum-free culture medium and seeded in transwell inserts. After 6 Gy radiation, complete culture medium containing with or without Fe-VO_x_ (50 μg/mL or 100 μg/mL) was added to the lower wells. The migrated cells in the bottom compartment were stained with crystal violet after incubation at 37°C for 12 hours. For the wound scratch assay, EC was seeded in a 6-well plate at 80% confluence. After 6 Gy radiation, Fe-VO_x_ (50 μg/mL or 100 μg/mL) was added and a sterile 200 μL pipette tip was used to form an artificial wound on the cell monolayer. Subsequently**,** the suspended cells were washed thoroughly with PBS and the wounds were observed at 0 or 12 hours. Relative migration rate (%) were quantified using ImageJ software.

**RNA sequencing.** Total RNA was extracted from macrophage using the TRIzol reagent according to the manufacturer's protocol. RNA concentration and integrity were assessed using a NanoDrop spectrophotometer and Agilent 2100 Bioanalyzer, respectively. High-quality RNA samples (RIN > 7.0) were selected for sequencing. Library preparation was performed using the Illumina TruSeq RNA Sample Preparation Kit, followed by sequencing on the Illumina NovaSeq platform to generate 150 bp paired-end reads. Raw sequencing data were processed using FastQC for initial quality control. Low-quality reads and adapter sequences were removed using Trimmomatic. The cleaned reads were then aligned to the mouse reference genome (GRCm38) using HISAT2. Gene expression levels were quantified using featureCounts, and differential gene expression analysis was conducted using DESeq2. Genes with an adjusted p-value (Padj) < 0.05 and |log_2_ fold change (log_2_FC)| > 1 were considered significantly differentially expressed.

Functional annotation of DEGs was performed using GO analysis, focusing on biological processes, cellular components, and molecular functions. KEGG pathway analysis was conducted to identify significant pathways associated with DEGs. The GO and KEGG analyses were performed by R packages such as clusterProfiler^4^. GSEA was performed using the GSEA software to evaluate the enrichment of specific gene sets in the context of Fe-VO_x_ treatment. The analysis was conducted with the MSigDB gene sets database. The PPI network of DEGs was constructed using the STRING database^5^. Interactions with a combined score > 0.4 were considered significant. The PPI network was visualized using Cytoscape software, and key hub genes within the network were identified using the CytoHubba plugin.

**IF staining.** Macrophage, EC and HBMSC were cultured on coverslips at 80% confluence. After 6 Gy radiation, cells were treated with Fe-VO_x_ (50 μg/mL or 100 μg/mL) for 48 h. Experimental procedures were performed as previously described^2^. The nuclei were stained with 4’,6-diamidino-2-phenylindole (DAPI). Images were visualized under an inverted fluorescence microscope (Leica Microsystems, Mannheim, Germany) and quantified using ImageJ software. The antibodies used are listed in Table S2.

**Animals and treatment.** Male SD rats aged 6-8 weeks were provided by the Animal Core Facility of Nanjing Medical University. The rats were separated into five groups (*n* = 5): control, 40Gy+*S.aureus*, 40Gy+*S.aureus*+NIR, 40Gy+*S.aureus*+Fe-VO_x_ (100 μg/mL), and 40Gy+*S.aureus*+Fe-VO_x_ (100 μg/mL) +NIR, respectively. A medical RS2000 irradiator described above was applied for radiation delivery. The measurements were taken 10 cm away from the source with a 2 × 2 cm^2^ radiation field with an incident beam on the left mandibles. A total dose of 40 Gy (8 Gy once daily for 5 consecutive days, dose rate = 1.265 Gy·min ^-1^) was delivered under general anesthesia following intraperitoneal injection of ketamine (100 mg/kg). At 10 days after the last radiation, the first and second molars of the mandible were extracted. Subsequently**,** *S. aureus* was topically implanted into the TESs. NIR laser exposing and Fe-VO_x_ (100 μg/mL) injecting were administered into the TESs every 3 days. At 14 days after extraction, bacteria were collected from the surface of the jawbone for plate smearing to estimate the *in vivo* antibacterial efficacy. Meanwhile, the clinical appearance of the gingival mucosa was observed and mandibles were harvested and fixed with 4% paraformaldehyde.

***In vivo* biodistribution of Fe-VO_x_.** After administrations of Fe-VO_x_ rats were sacrificed at 6, 12, 24, and 48 h post injection. Major organs (heart, liver, spleen, lung, and kidneys) were dissected, rinsed with PBS, weighted. The content of V in the samples was analyzed using ICP-MS.

**Micro-CT analysis.** Micro-CT (Skyscan 1176, Kontich, Belgium) scans were used for analysis as previous described^6^. The regions of interest were defined to focus on the TESs of the rat mandibles and analyzed by the following parameter: bone volume over tissue volume (BV/TV).

**Histological analysis.** Human mandible samples from both healthy individuals and ORNJ patients, along with rat mandible samples were harvested. Subsequently, samples were decalcified in 10% ethylenediaminetetraacetic acid (EDTA) and 4 μm-thick paraffin-embedded sections were prepared for HE, Masson’s trichrome, and IF staining as previous described^1^. The antibodies used are listed in Table S2.

**Statistical analysis.** Statistical analysis were performed by GraphPad Prism 8.0.1 (GraphPad Software,CA, USA). Filter and standardize the data before statistical analysis. The differences in means between different groups were compared *via* Student’s t-test or one-way analysis of variance (ANOVA). All sample sizes/experiments are repeated at least 3 times. The significant differences were determined by ^**^P < 0.01, ^*^P < 0.05, ^##^P < 0.01, ^#^P < 0.05, ^@@^P < 0.01, ^@^P < 0.05 and ^¥¥^P < 0.01.

**References**

(1) Qin, Z.; Han, Y.; Du, Y.; Zhang, Y.; Bian, Y.; Wang, R.; Wang, H.; Guo, F.; Yuan, H.; Pan, Y.; et al. Bioactive materials from berberine-treated human bone marrow mesenchymal stem cells promote alveolar bone regeneration by regulating macrophage polarization. *Sci China Life Sci* **2024**, *67* (5), 1010-1026.

(2) Wang, Y.; Yuan, Y.; Wang, R.; Wang, T.; Guo, F.; Bian, Y.; Wang, T.; Ma, Q.; Yuan, H.; Du, Y.; et al. Injectable Thermosensitive Gel CH-BPNs-NBP for Effective Periodontitis Treatment through ROS-Scavenging and Jaw Vascular Unit Protection. *Adv Healthc Mater* **2024**, *13* (22), e2400533.

(3) Feng, Y.; Luo, X.; Li, Z.; Fan, X.; Wang, Y.; He, R. R.; Liu, M. A ferroptosis-targeting ceria anchored halloysite as orally drug delivery system for radiation colitis therapy. *Nat Commun* **2023**, *14* (1), 5083.

(4) Yu, G.; Wang, L. G.; Han, Y.; He, Q. Y. clusterProfiler: an R package for comparing biological themes among gene clusters. *OMICS* **2012**, *16* (5), 284-287.

(5) Szklarczyk, D.; Kirsch, R.; Koutrouli, M.; Nastou, K.; Mehryary, F.; Hachilif, R.; Gable, A. L.; Fang, T.; Doncheva, N. T.; Pyysalo, S.; et al. The STRING database in 2023: protein-protein association networks and functional enrichment analyses for any sequenced genome of interest. *Nucleic Acids Res* **2023**, *51* (D1), D638-D646.

(6) Guo, F.; Li, J.; Chen, Z.; Wang, T.; Wang, R.; Wang, T.; Bian, Y.; Du, Y.; Yuan, H.; Pan, Y.; et al. An Injectable Black Phosphorus Hydrogel for Rapid Tooth Extraction Socket Healing. *ACS Appl Mater Interfaces* **2024**, *16* (20), 25799-25812.


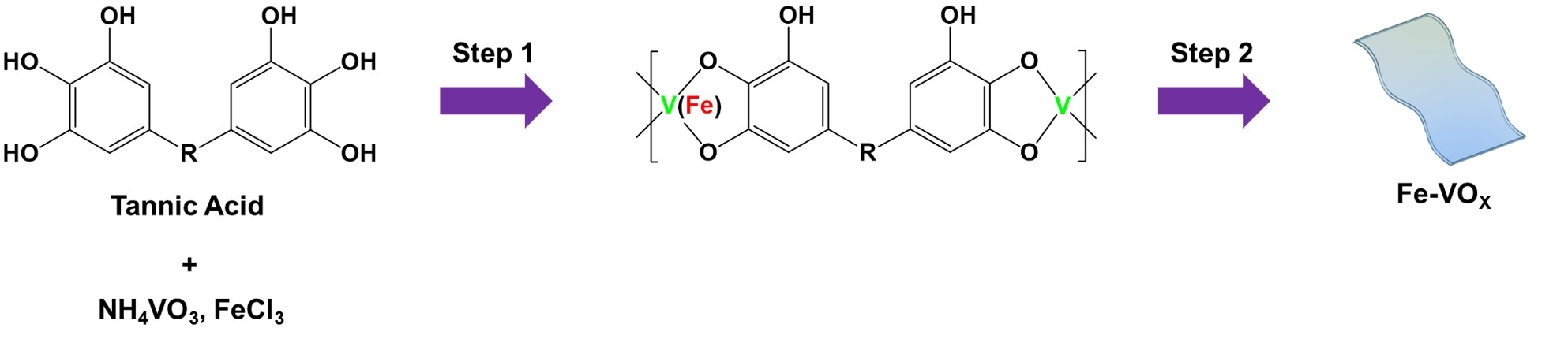


**Figure S1.** Schematics of the synthesis of Fe-VOx nanozymes.


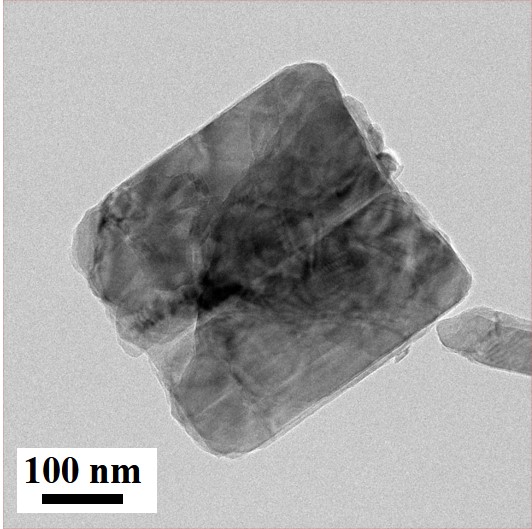


**Figure S2.** TEM image of VO_x_.


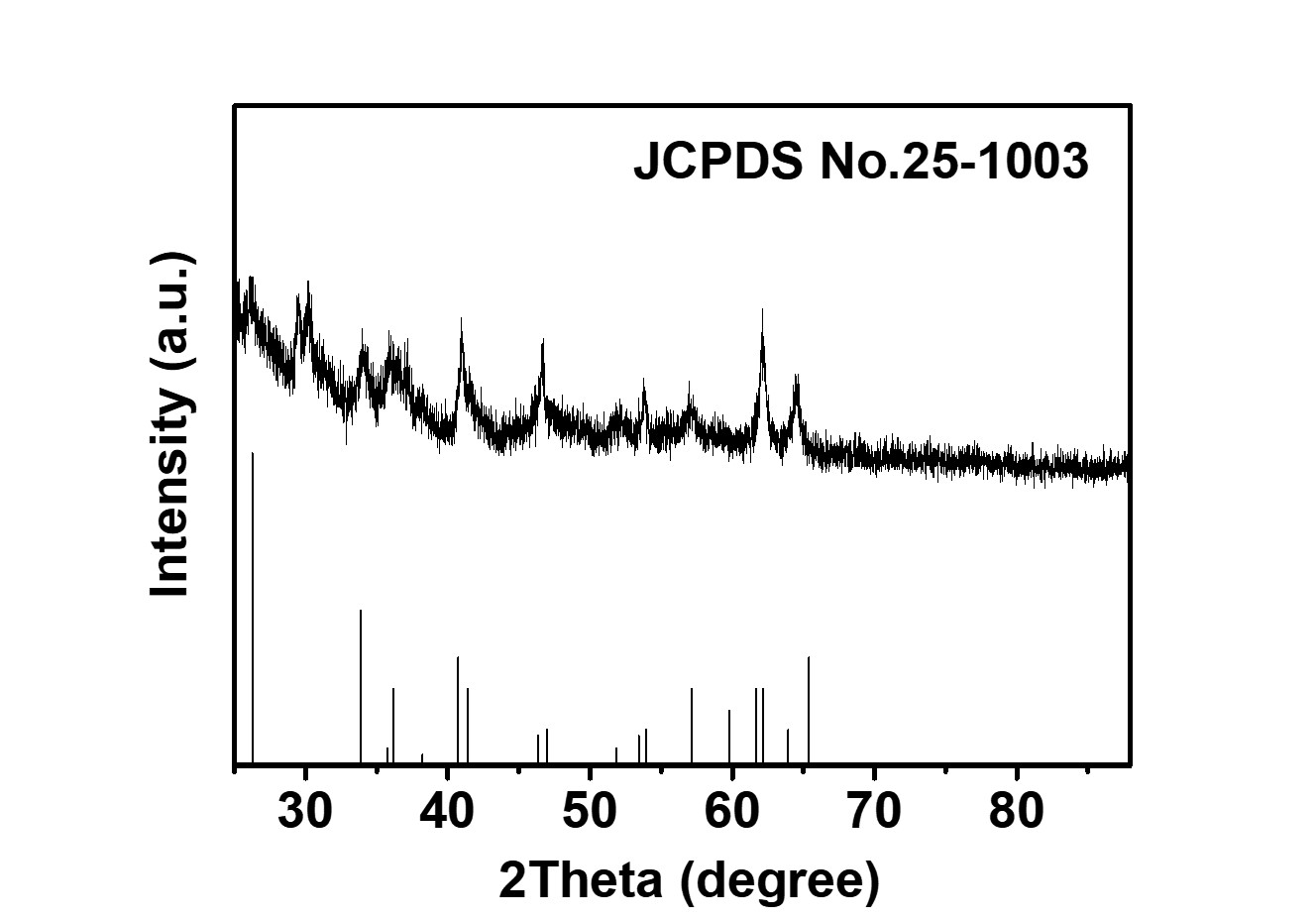


**Figure S3.** XRD pattern of Fe-VO_x_ nanozymes.


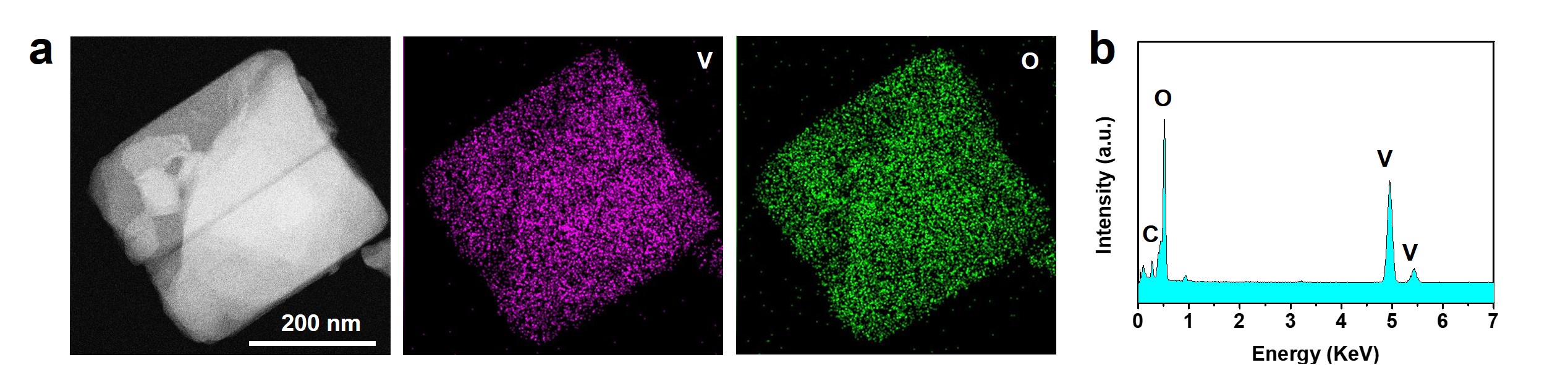


**Figure S4. (a)** EDS elemental mapping images and of VO_x_ nanozymes. **(b)** EDS spectrum of VO_x_.


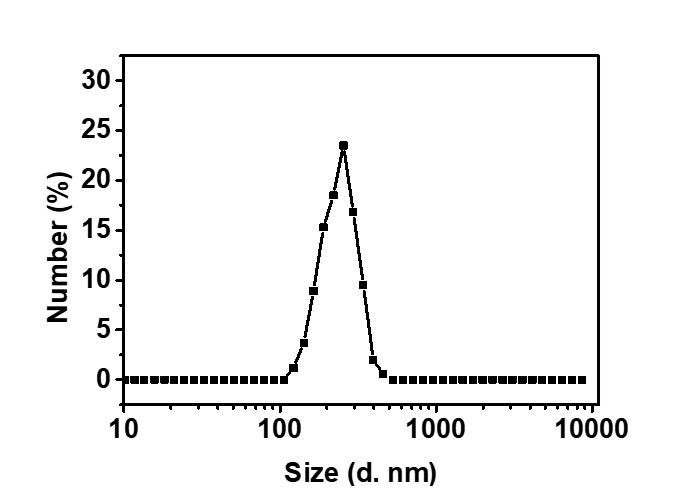


**Figure S5.** Size distribution of VO_x_.


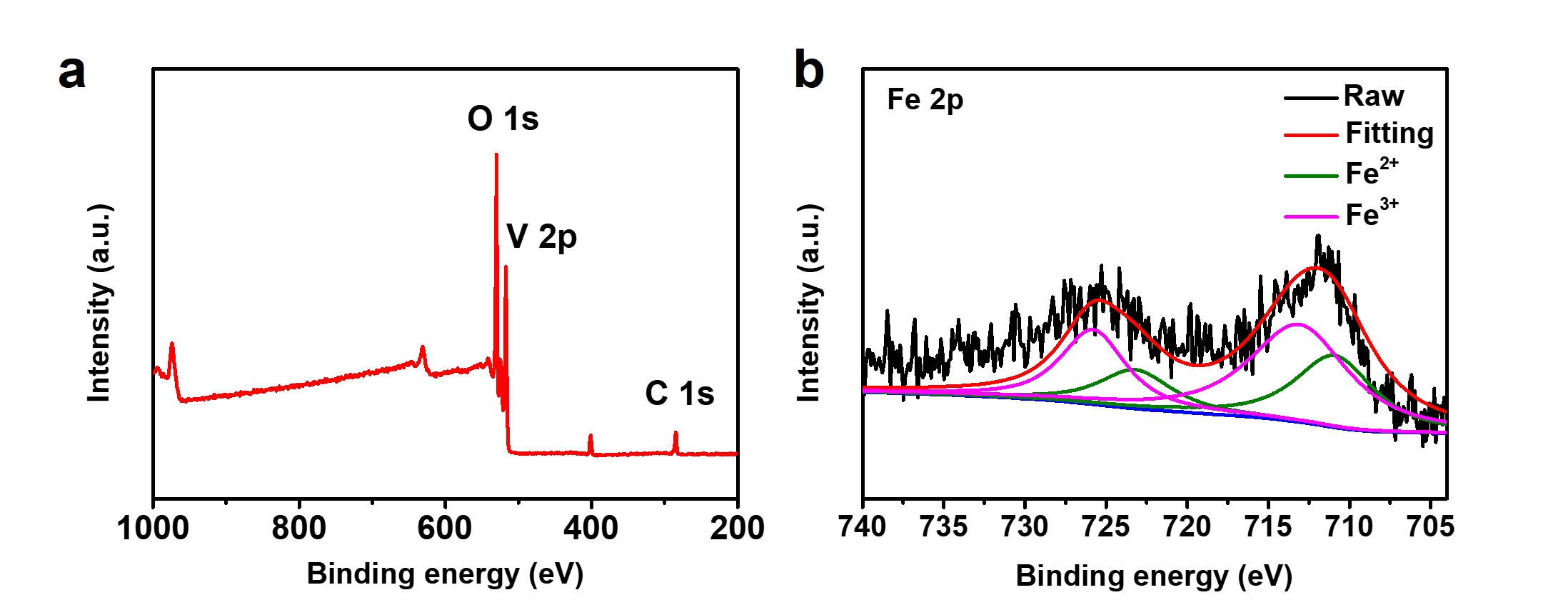


**Figure S6.** **(a)** XPS spectrum of VO_x_. **(b)** High-resolution scans of Fe 2p of Fe-VO_x_.


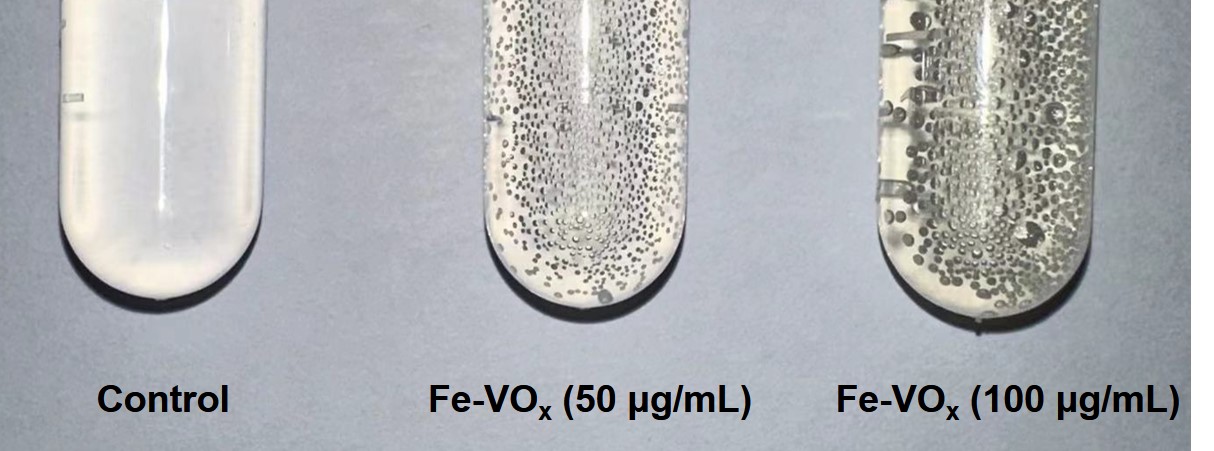


**Figure S7.** Digital picture of H_2_O_2_ solutions with different samples.


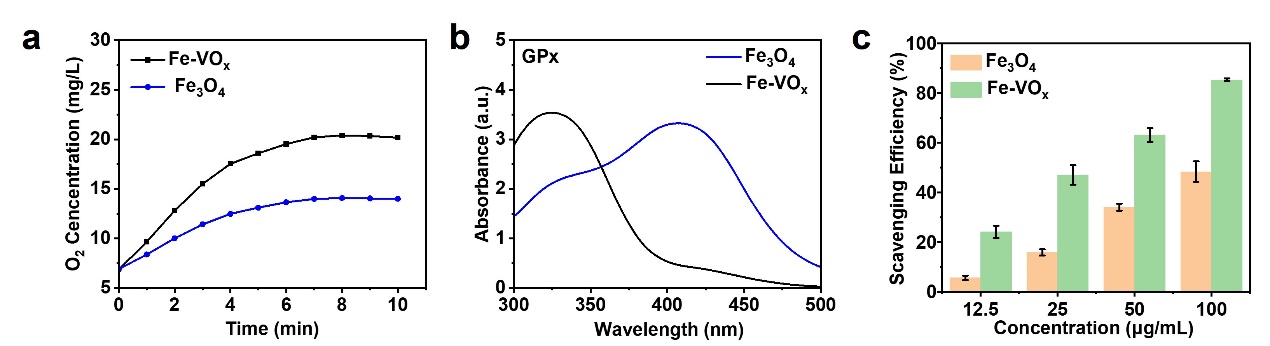


**Figure S8. (a)** O_2_ concentration of H_2_O_2_ solution with different samples (100 μg/mL). **(b)** DTNB absorption with Fe_3_O_4_ and Fe-VO_x_. **(c)** SOD-like activity of Fe_3_O_4_ and Fe-VO_x_ at different concentrations.


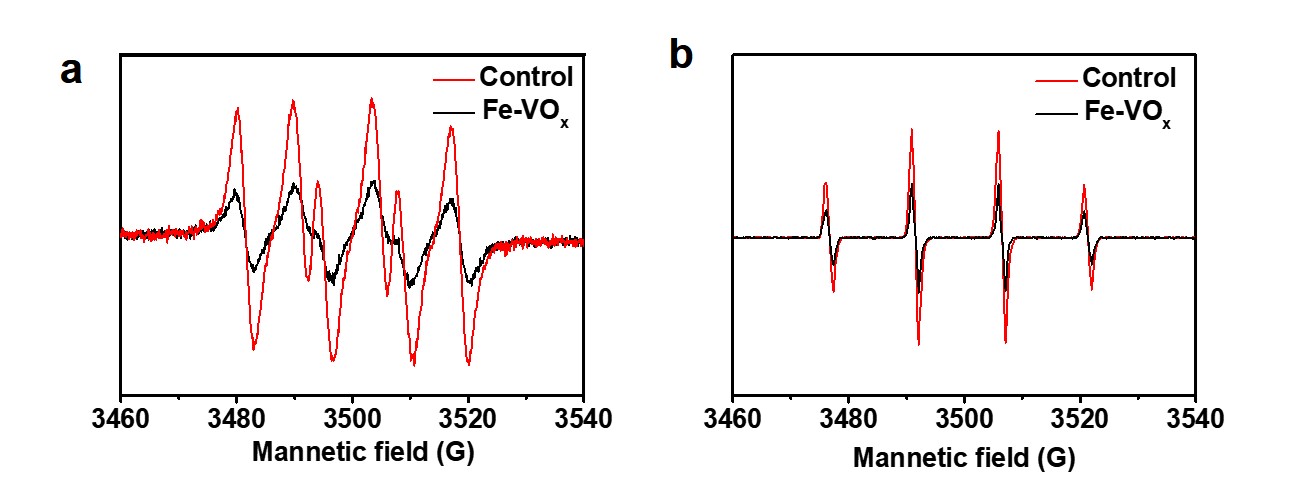


**Figure S9. (a)** ESR spectra for •O_2_^-^ scavenging property of Fe-VO_x_. **(b)** ESR spectra for •OH scavenging property of Fe-VO_x_.


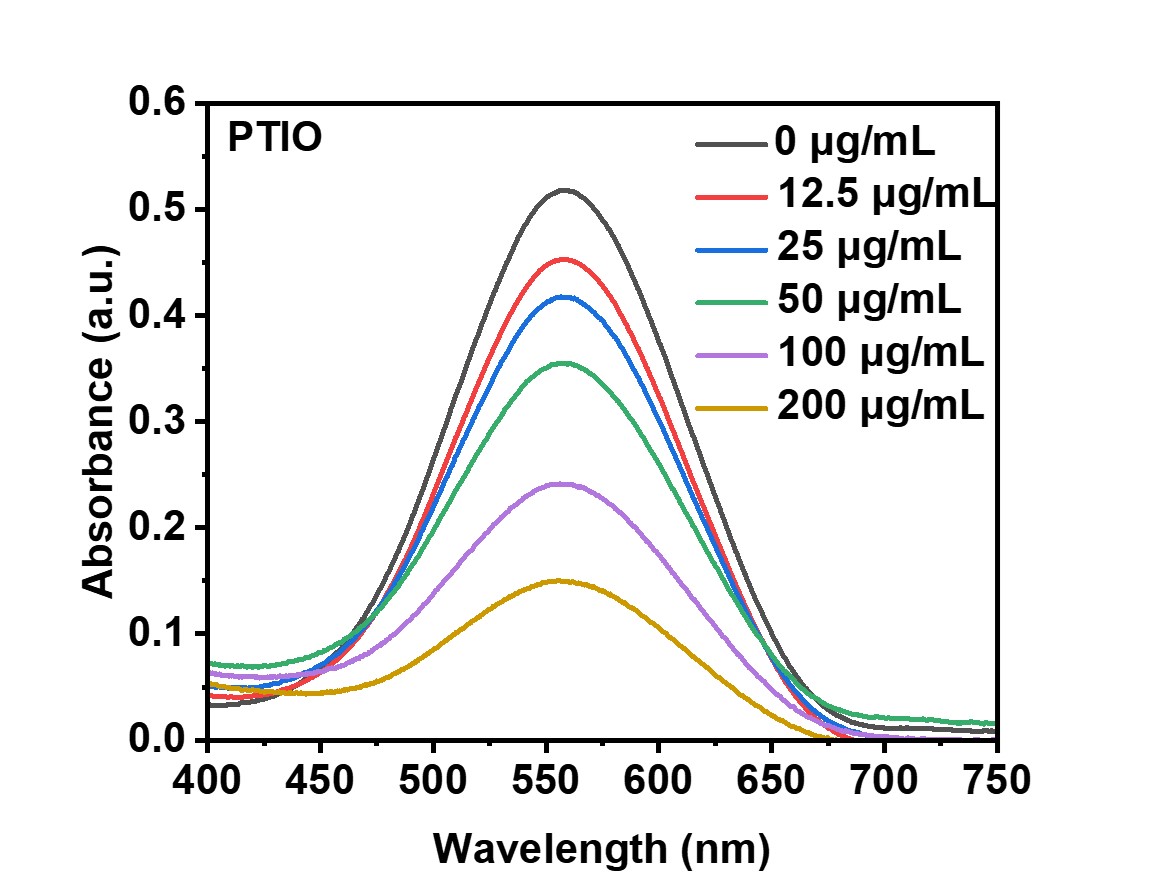


**Figure S10.** PTIO absorption with Fe-VO_x_ nanozymes of different concentrations.


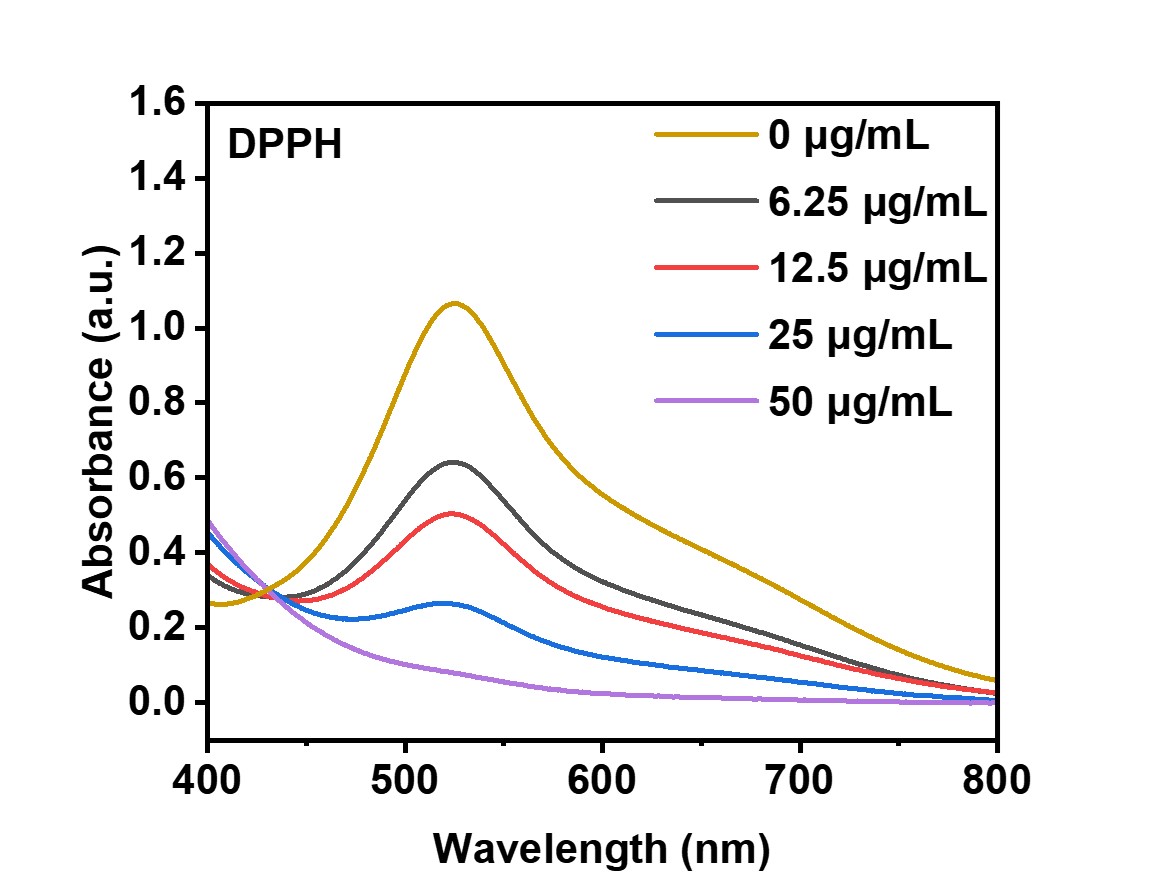


**Figure S11.** DPPH absorption with Fe-VO_x_ nanozymes of different concentrations.


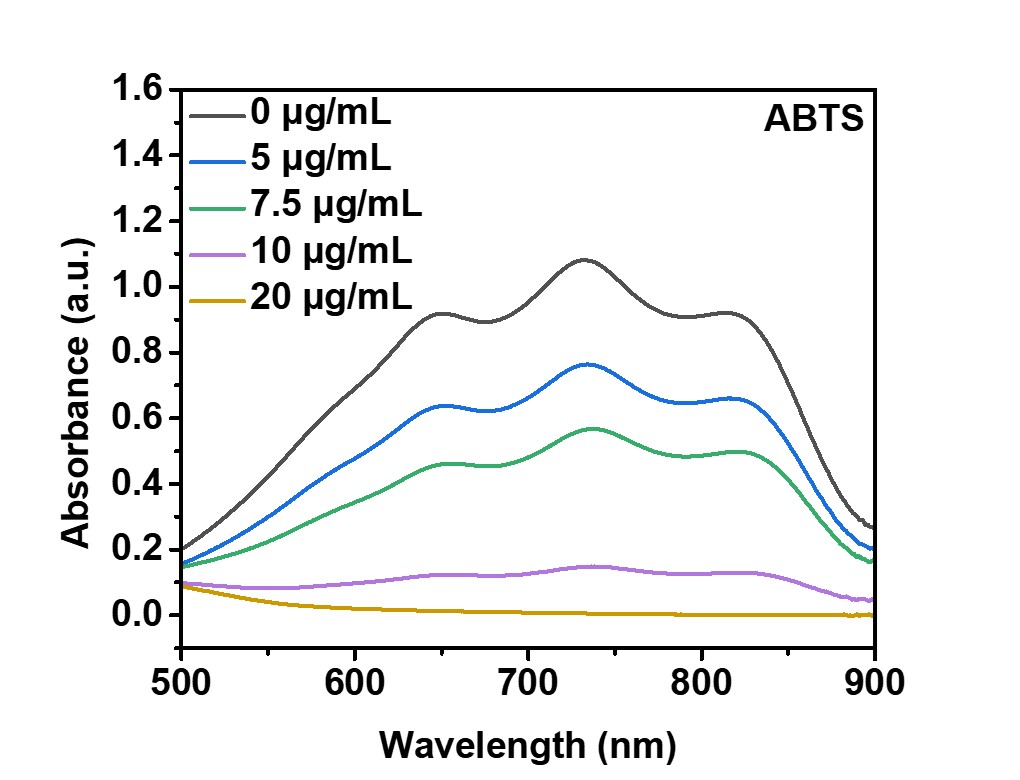


**Figure S12.** ABTS absorption with Fe-VO_x_ nanozymes of different concentrations.


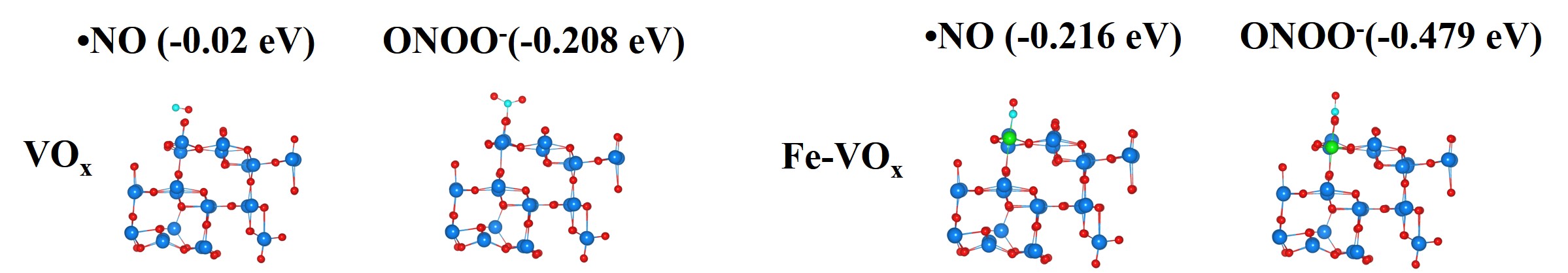


**Figure S13.** Energy feature of the segment model with different radical units.


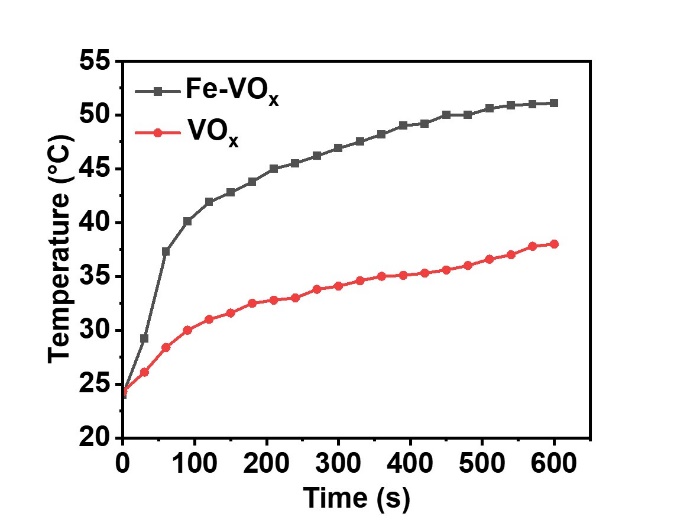


**Figure S14.** Temperature variation curve of different samples (100 μg/mL) after 808 nm laser (1.5 W/cm^2^) irradiation.


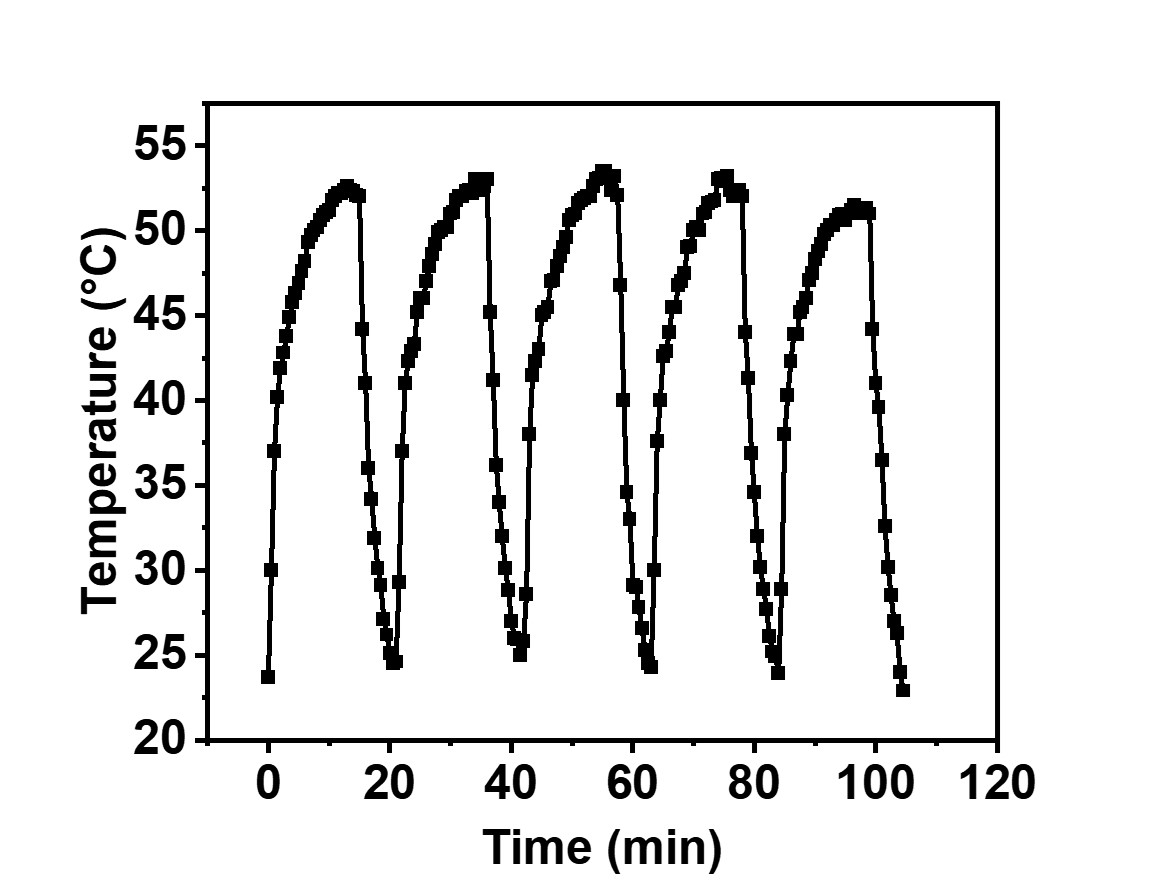


**Figure S15.** Reversible photothermal cycling properties of Fe-VO_x_.


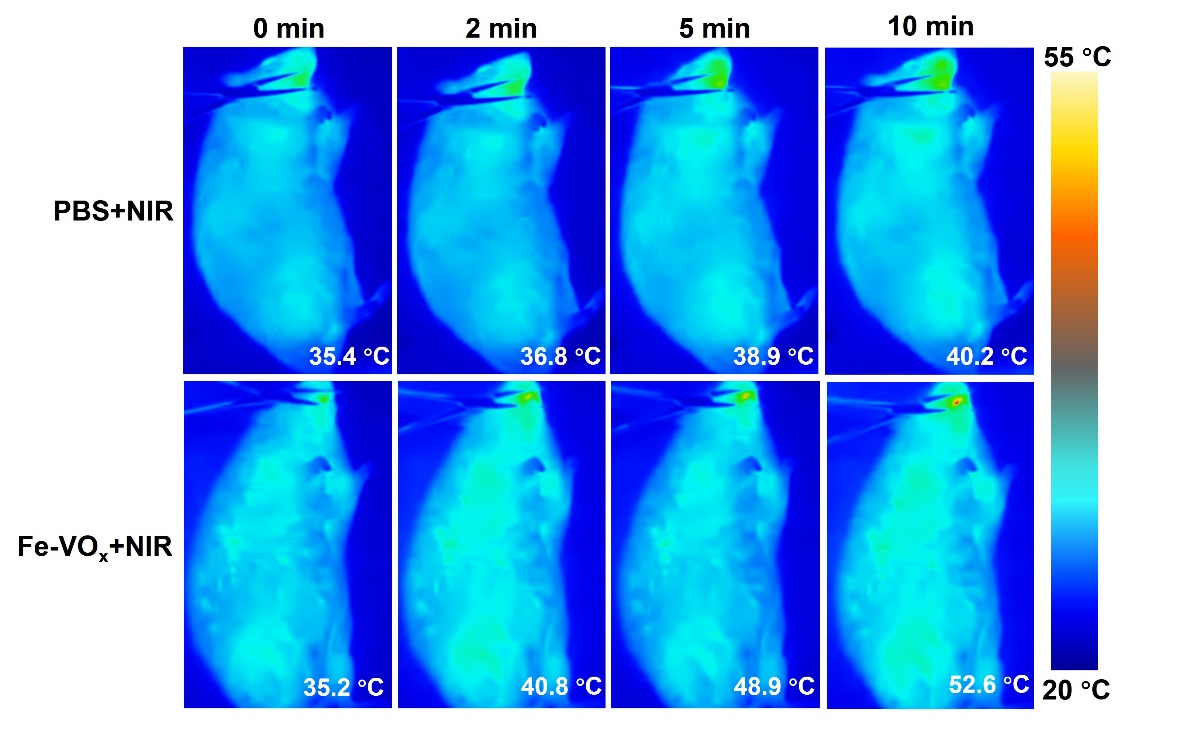


**Figure S16.** *In vivo* thermal images of SD rat in the groups of PBS, Fe-VO_x_ during NIR irradiation at different time intervals.


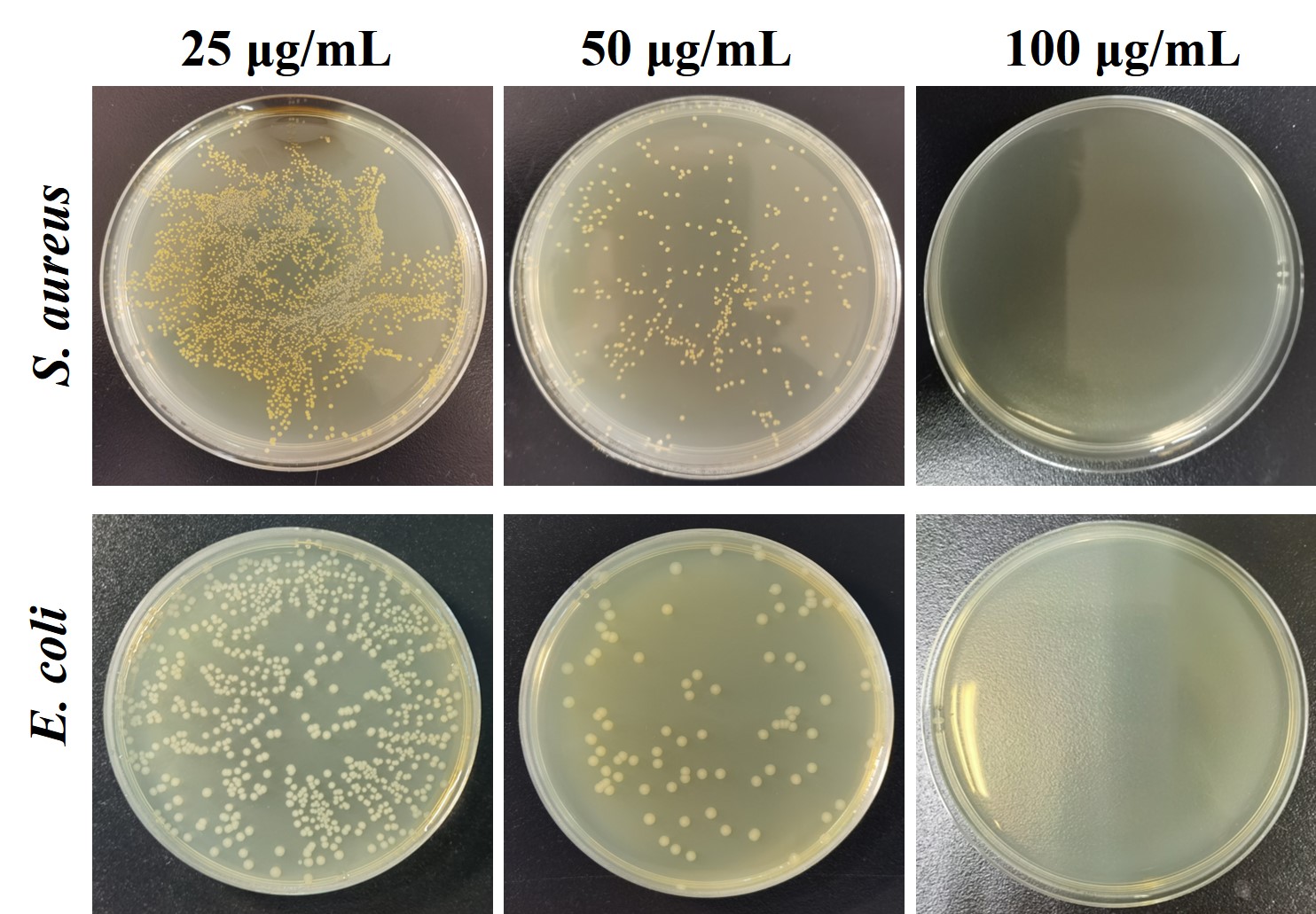


**Figure S17.** The antibacterial effect of PTT-induced by Fe-VO_x_ (NIR laser power, 1.5 W/cm^2^) at different concentrations on *S. aureus* and *E. coli* was evaluated by colony counting.


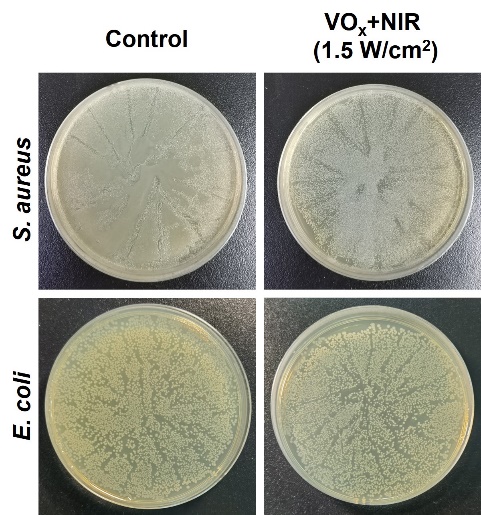


**Figure S18.** Photothermal antibacterial effect of VO_x_ on *S. aureus* and *E. coli* evaluated by colony counting.


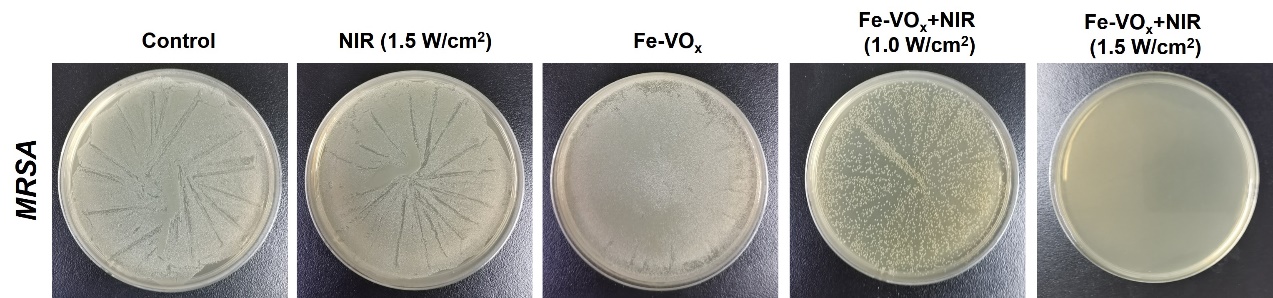


**Figure S19.** The antibacterial effect of the different treatments on *MRSA* was evaluated by colony counting.

**
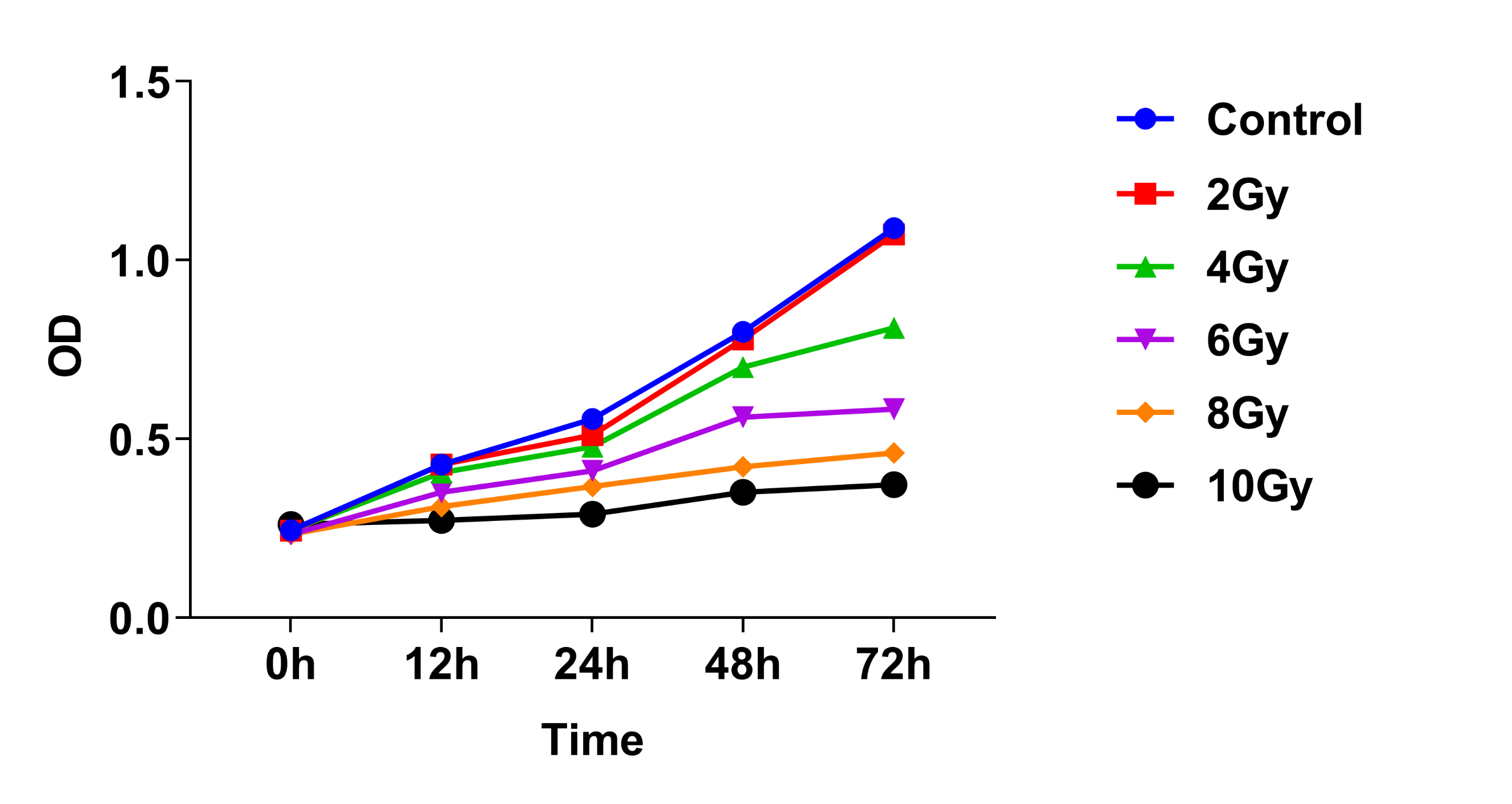
**

**Figure S20.** Cell viabilities of macrophage treated with increasing radiation doses. (n=3).


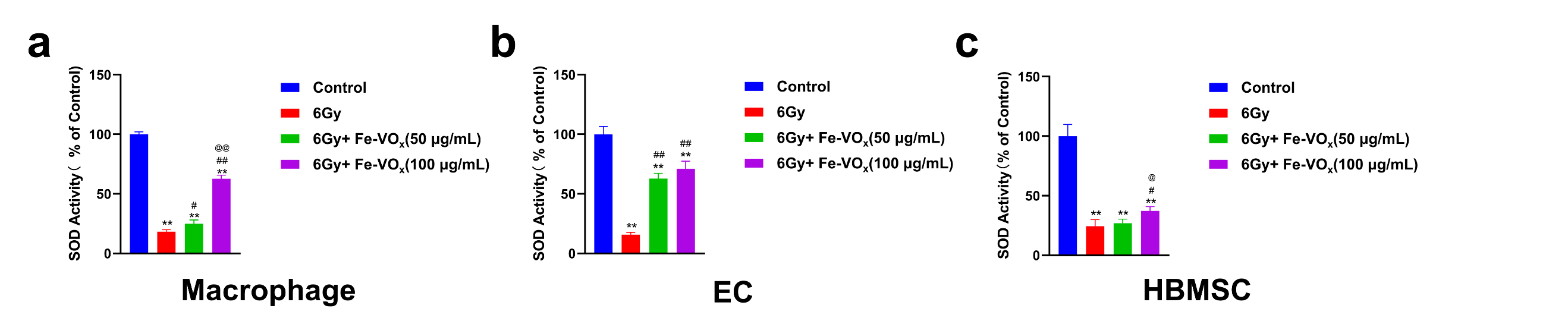


**Figure S21.** SOD activity assessment of **(a)** macrophage, **(b)** EC and **(c)** HBMSC with or without Fe-VO_x_ (50 or 100 μg/mL) treatment after 6Gy radiation at 48 h, respectively. ^**^P < 0.01 versus the control group, ^##^P < 0.01 and ^#^P < 0.05 versus the 6Gy group, ^@@^P < 0.01 and ^@^P < 0.05 versus the 6Gy +Fe-VO_x_ (50 μg/mL) group. (n=3).


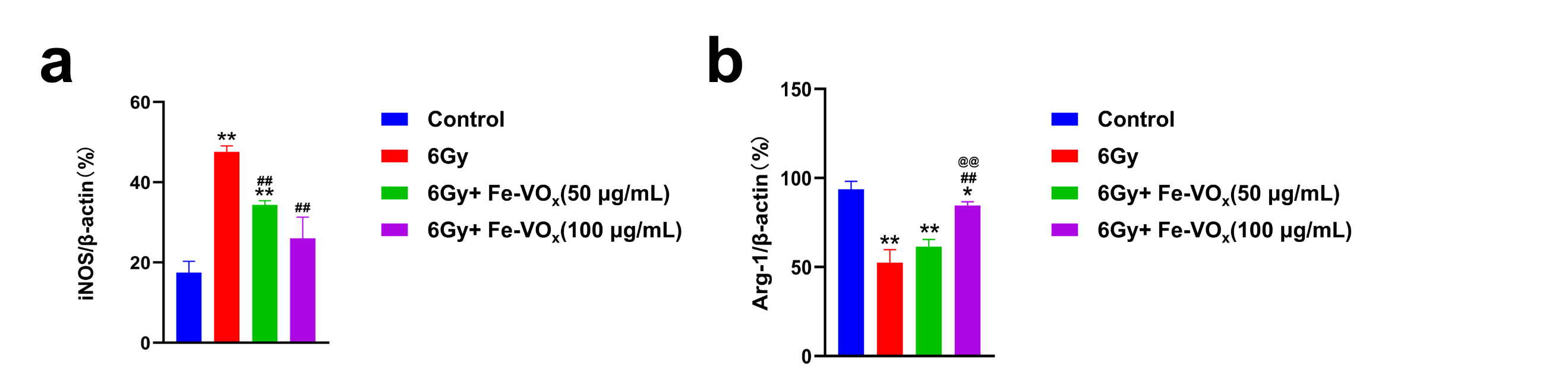


**Figure S22.** Quantification analysis of the western blot analysis of macrophage with or without Fe-VO_x_ (50 or 100 μg/mL) treatment after 6 Gy radiation at 48 h, ^**^P < 0.01 and ^*^P < 0.05 versus the control group, ^##^P < 0.01 versus the 6Gy group, ^@^P < 0.05 versus the 6Gy +Fe-VO_x_ (50 μg/mL) group. (n=3).

^
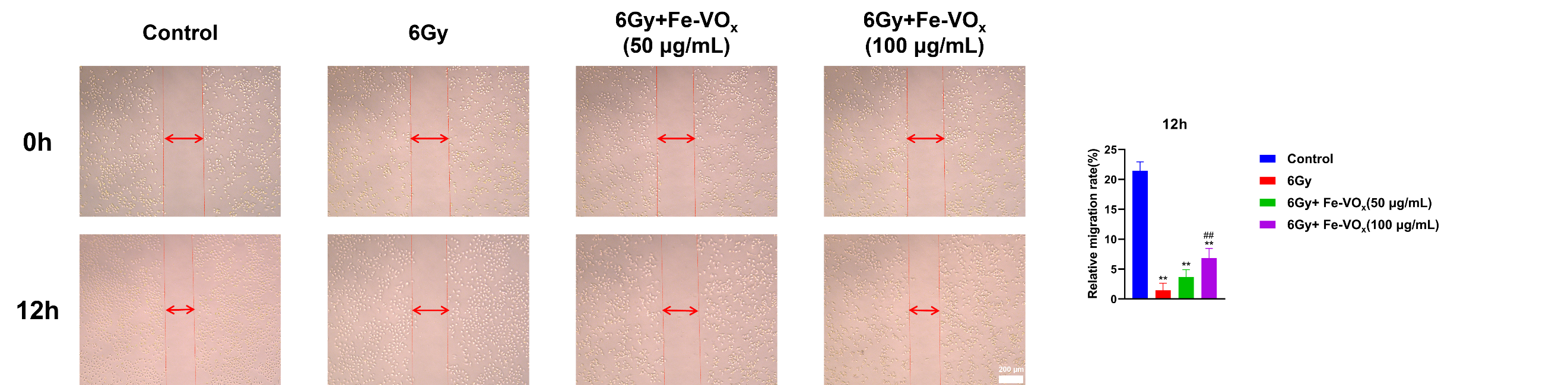
^

**Figure S23.** Representative images and quantification analysis of wound scratch assay of EC with or without Fe-VO_x_ (50 or 100 μg/mL) treatment after 6Gy radiation at 0 and 48 h, scale bar=200 µm, ^**^P < 0.01 versus the control group, ^#^P < 0.05 versus the 6Gy group. (n=3).


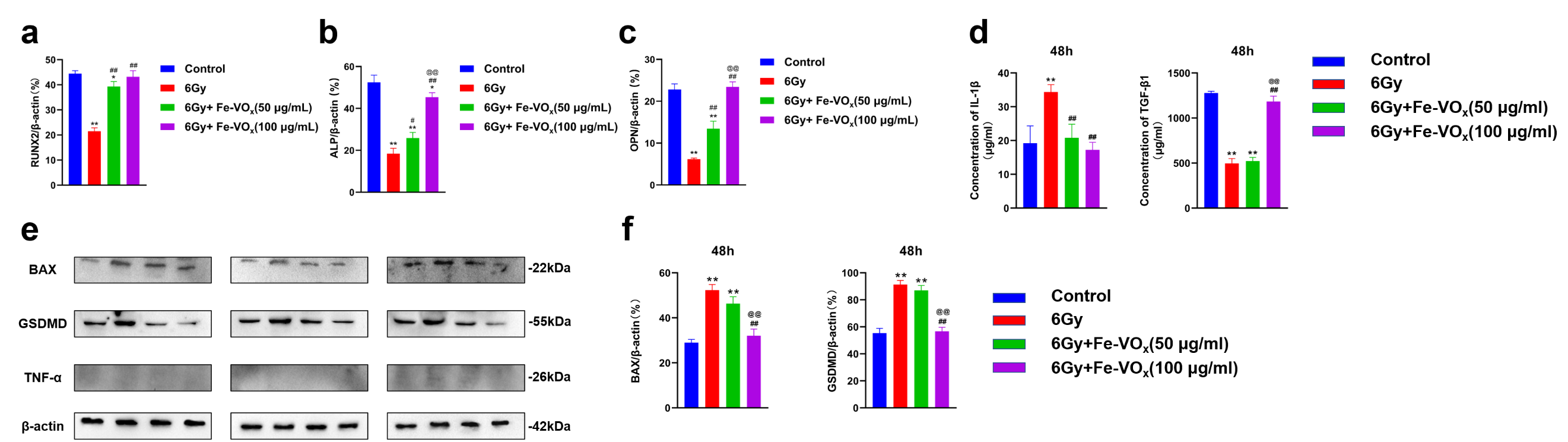


**Figure S24.** **(a-c)** Quantification analysis of the western blot analysis of HBMSC with or without Fe-VO_x_ (50 or 100μg/mL) treatment after 6Gy radiation on day 3, **(d)** Levels of IL-1β and TGF-β1 in macrophage’s supernatant were measured by ELISA, **(e, f)** Protein expressions and quantification analysis of BAX, GSDMD and TNF-α in macrophage were determined by western blot analysis. ^**^P < 0.01 and ^*^P < 0.05 versus the Control group, ^##^P < 0.01 and ^#^P < 0.05 versus the 6Gy group, ^@@^P < 0.01 versus the 6Gy +Fe-VO_x_ (50μg/mL) group. (n=3).


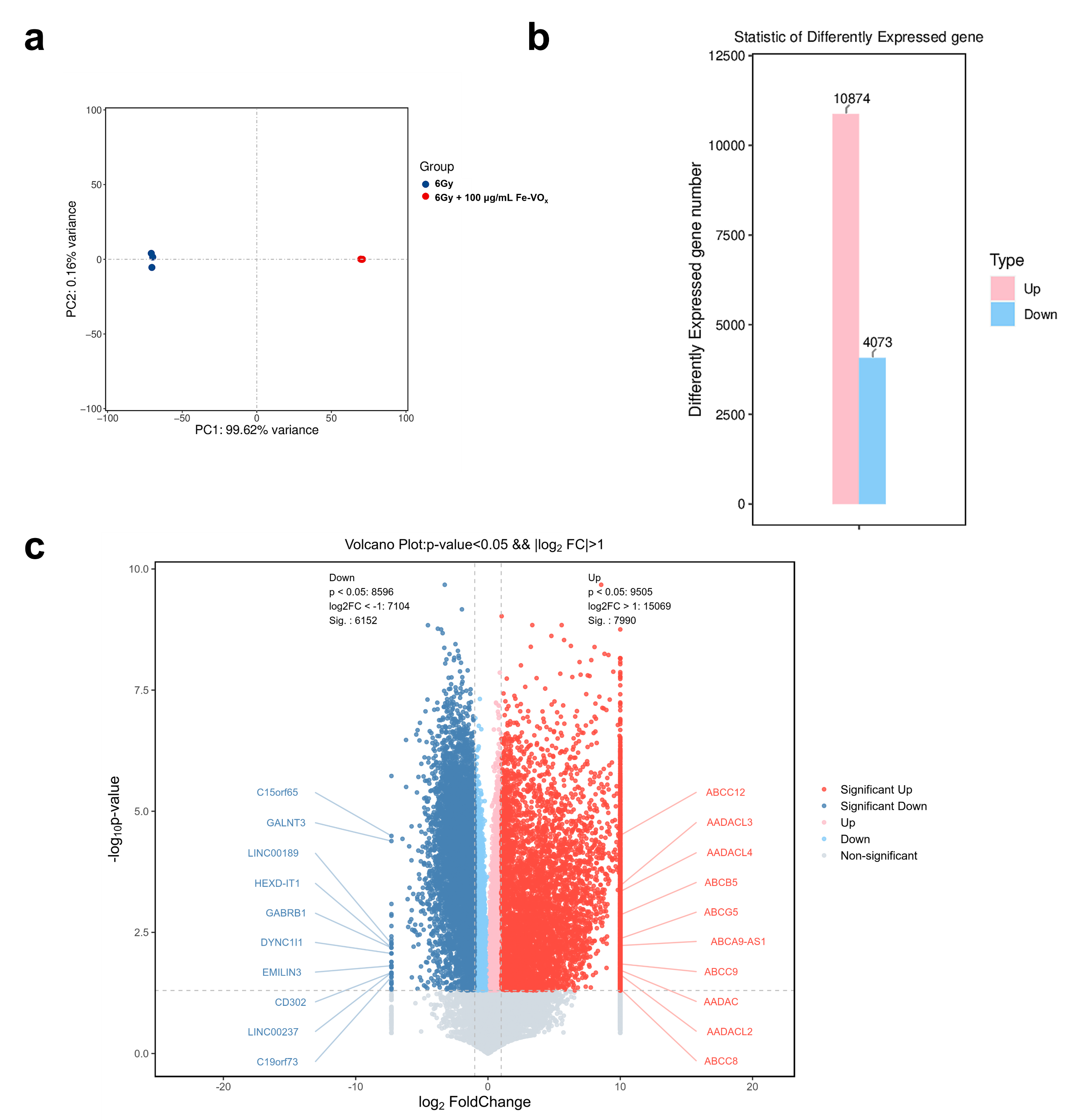


**Figure S25.** Differential gene expression analysis of 6Gy and 6Gy + 100 μg/mL Fe-VO_x_ Groups. **(a)** Principal Component Analysis (PCA) plot illustrating the variance in gene expression profiles between the 6Gy (blue) and 6Gy + 100 μg/mL Fe-VO_x_ (red) groups, **(b)** Bar chart showing the number of DEGs, and **(c)** Volcano plot displaying DEGs with a p-value < 0.05 and |log2 Fold Change| > 1. Notable genes with the most significant changes are highlighted on the plot.

**
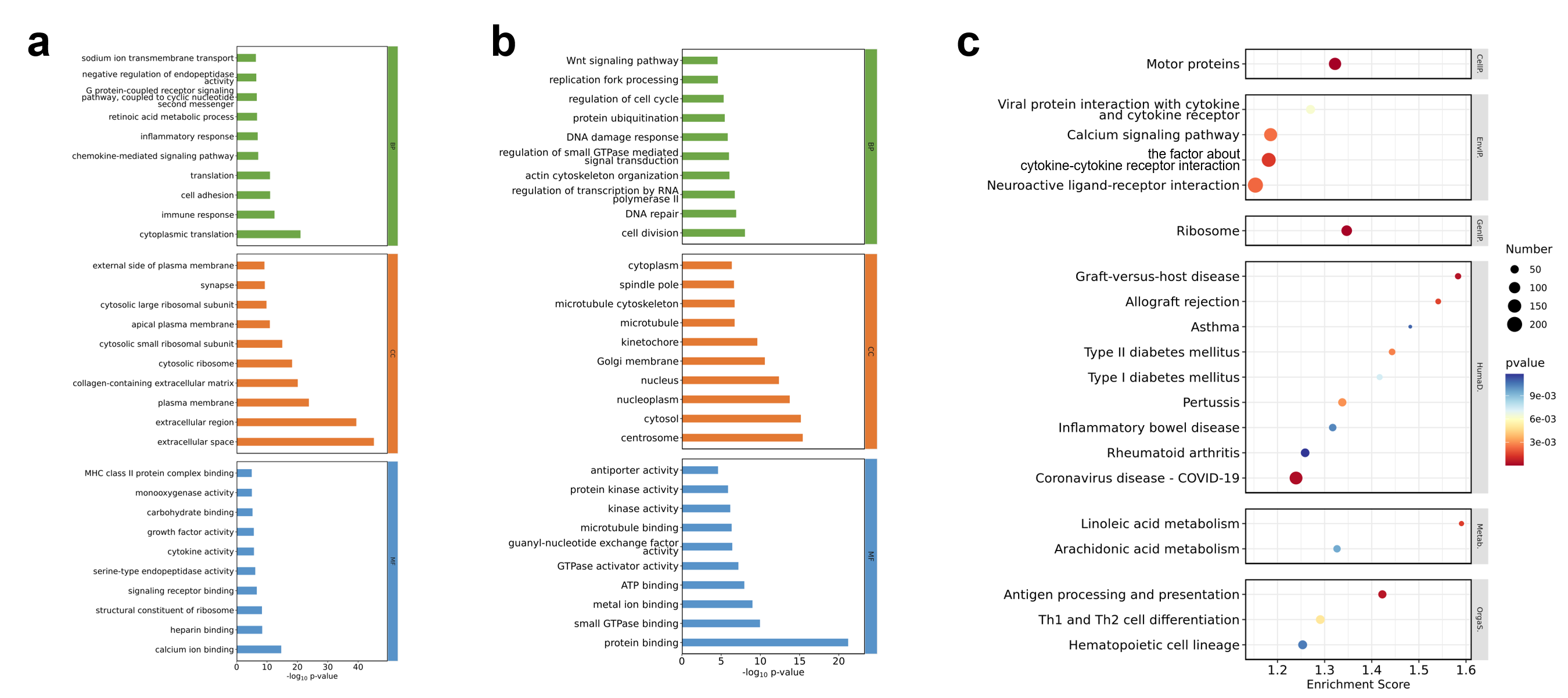
**

**Figure S26.** GO analysis of **(a)** upregulated genes and **(b)** downregulated genes in the 6Gy + 100 μg/mL Fe-VO_x_ group, highlighting the most significantly biological processes. **(c)** KEGG pathway analysis of all differentially expressed genes, identifying the key pathways enriched in the 6Gy + 100 μg/mL Fe-VO_x_ group.

**
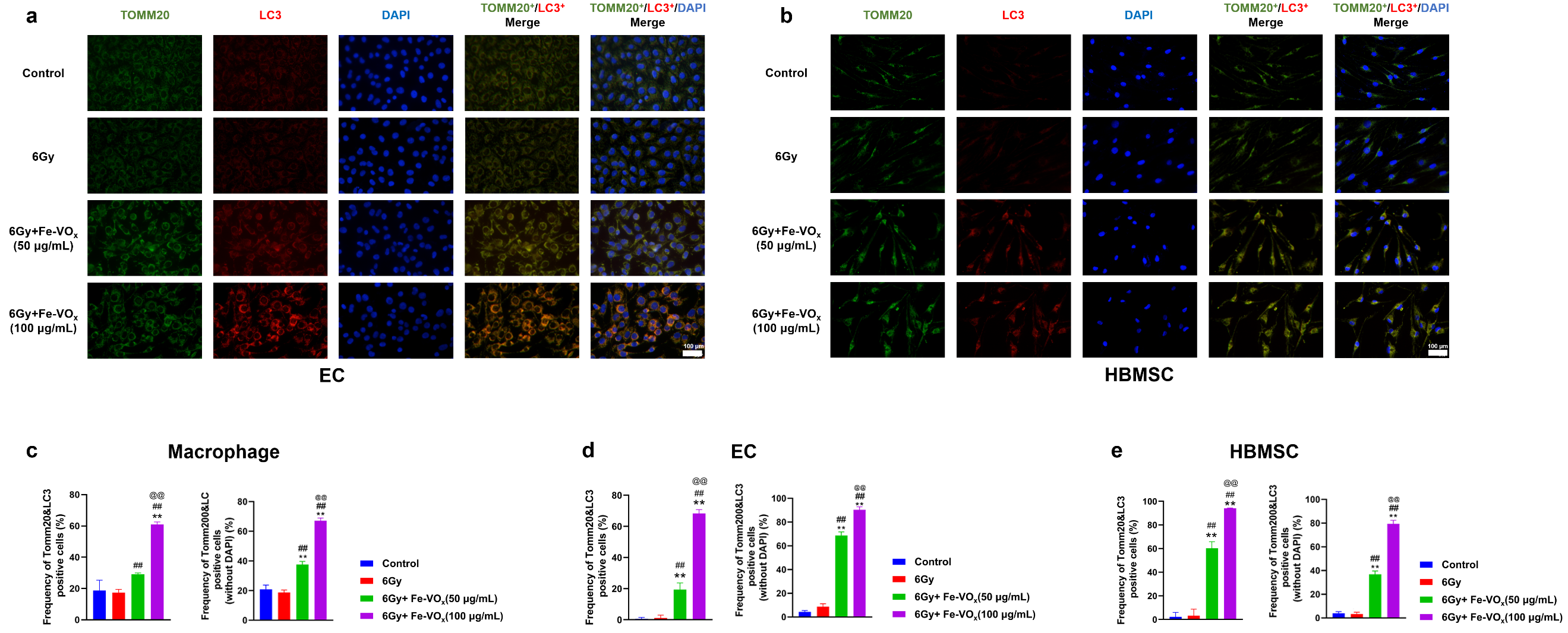
**

**Figure S27.** IF staining showing the TOMM20^+^/ LC3^+^ colocalization in **(a)** EC and **(b)** HBMSC with or without Fe-VO_x_ (50 or 100 μg/ml) treatment after 6Gy radiation. Quantification analysis of the frequency of TOMM20^+^/ LC3^+^ colocalization in **(c)** macrophage, **(d)** EC and **(e)** HBMSC with or without Fe-VO_x_ (50 or 100 μg/mL) treatment after 6Gy radiation at 48 h, respectively. ^**^P < 0.01 versus the control group, ^##^P < 0.01 versus the 6Gy group, ^@@^P < 0.01 versus the 6Gy +Fe-VO_x_ (50 μg/mL) group, scale bars,100 μm. (n=3).


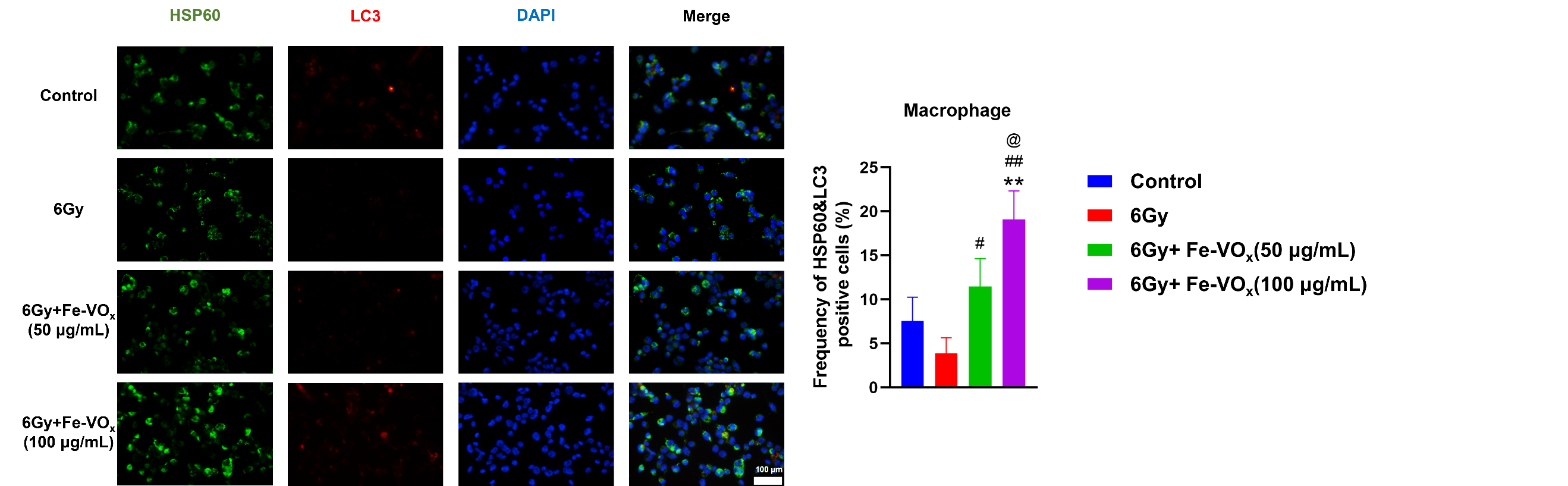


**Figure S28.** Representative images and quantification analysis of the frequency of HSP60^+^/ LC3^+^ colocalization in macrophage with or without Fe-VO_x_ (50 or 100 μg/mL) treatment after 6Gy radiation at 48h, scale bars, 100 μm. ^**^P < 0.01 versus the control group, ^##^P < 0.01 and ^#^P < 0.05 versus the 6Gy group, ^@^P < 0.05 versus the 6Gy +Fe-VO_x_ (50 μg/mL) group. (n=3).


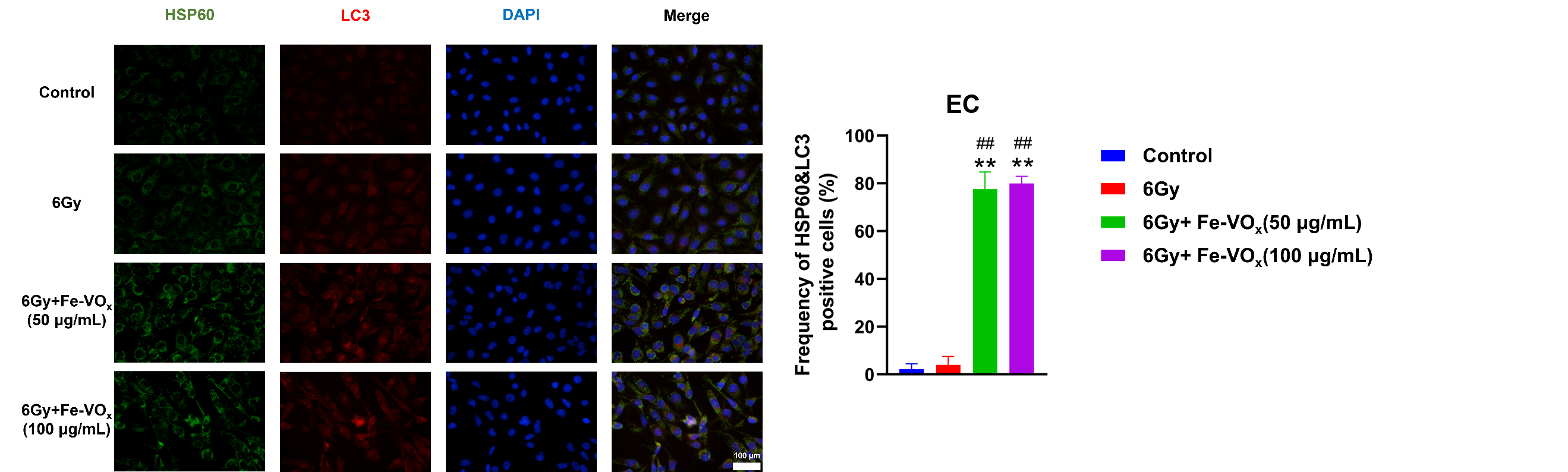


**Figure S29.** Representative images and quantification analysis of the frequency of HSP60^+^/ LC3^+^ colocalization in EC with or without Fe-VO_x_ (50 or 100 μg/mL) treatment after 6Gy radiation at 48 h, scale bars, 100 μm. ^**^P < 0.01 versus the control group, ^##^P < 0.01 versus the 6Gy group. (n=3).


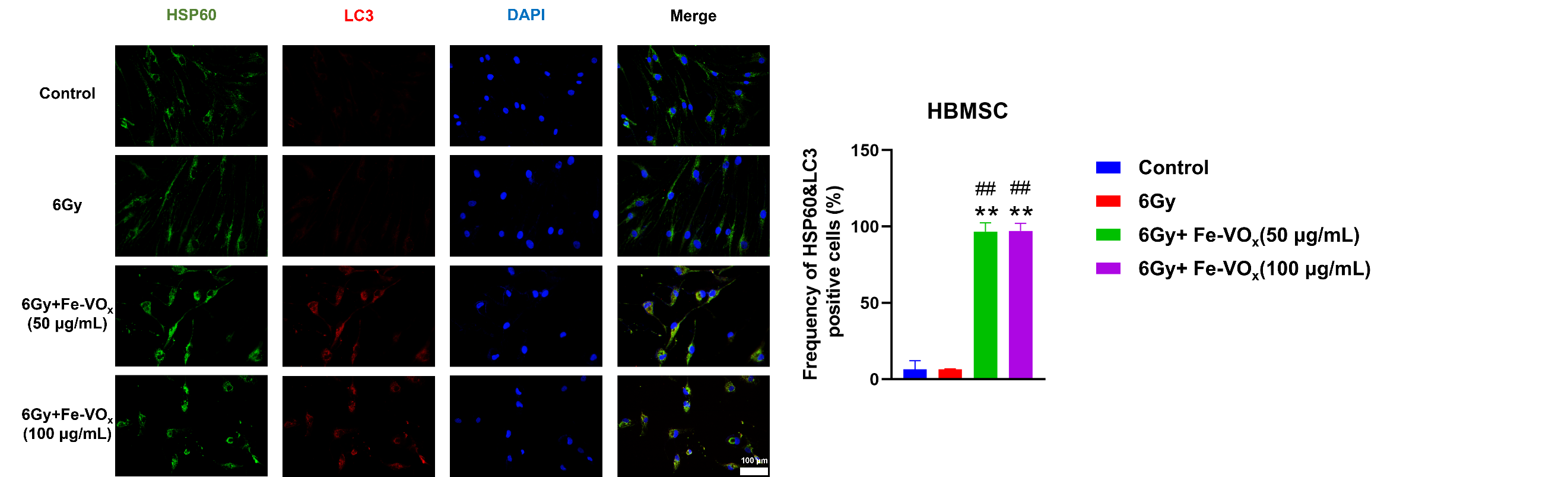


**Figure S30.** Representative images and quantification analysis of the frequency of HSP60^+^/ LC3^+^ colocalization in HBMSC with or without Fe-VO_x_ (50 or 100 μg/mL) treatment after 6Gy radiation at 48 h, scale bars, 100 μm. ^**^P < 0.01 versus the control group, ^##^P < 0.01 versus the 6Gy group. (n=3).


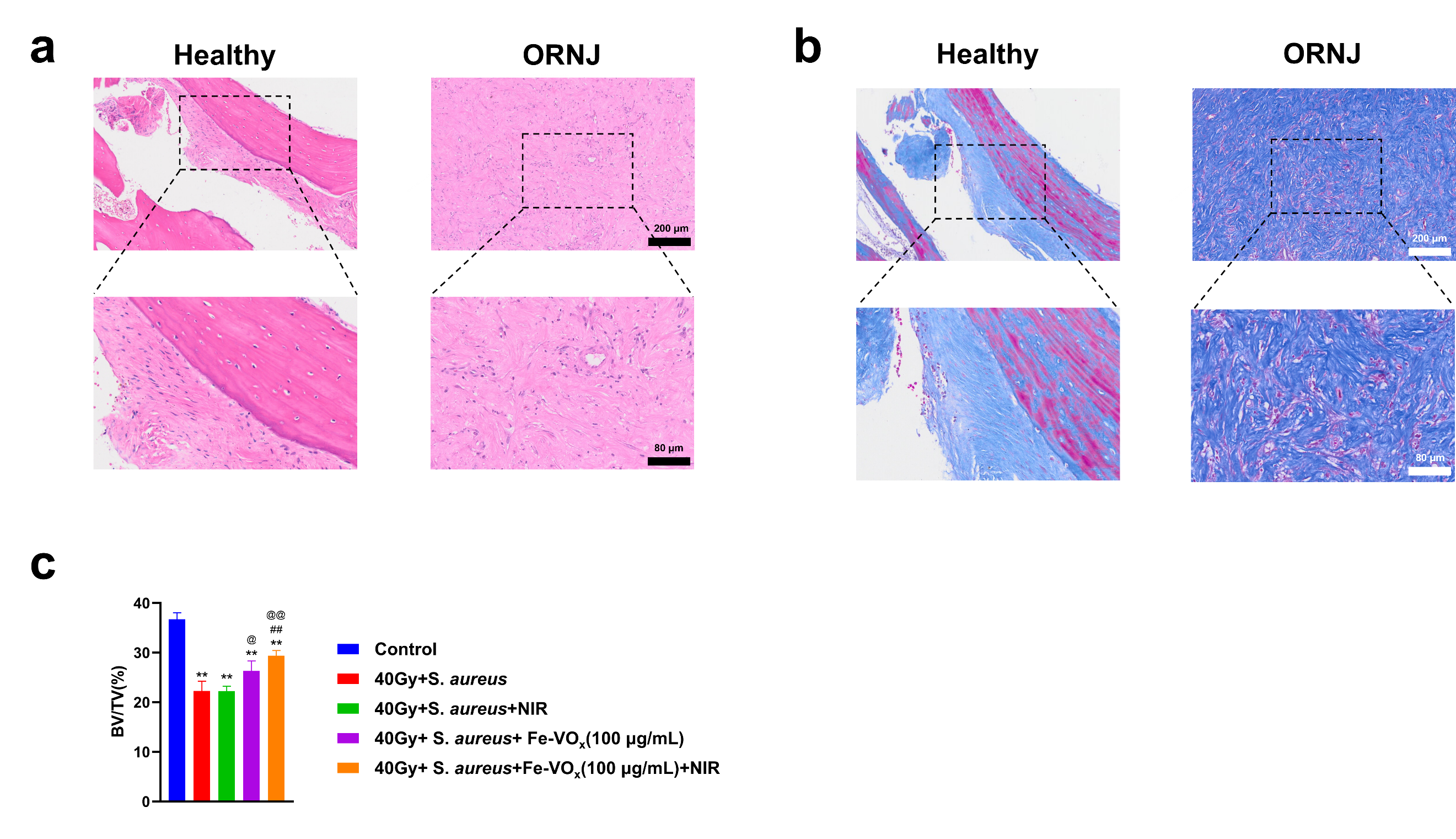


**Figure S31. (a)** HE staining of bone trabecula (black box) in mandible sections from both healthy individual and ORNJ patient, scale bars, 200 μm or 80 μm. **(b)** Masson’s staining of collagenous fibers (black box) in mandible sections from both healthy individual and ORNJ patient, scale bars, 200 μm or 80 μm. The experiment was repeated 3 times independently with similar results. **(c)** Quantification analysis of the BV/TV in TESs. ^**^P < 0.01 versus the control group, ^##^P < 0.01 versus the 40Gy+S. *aureus* group, ^@@^P < 0.01 and ^@^P < 0.05 versus the 40Gy+S. *aureus*+ NIR group. (n=3).

**
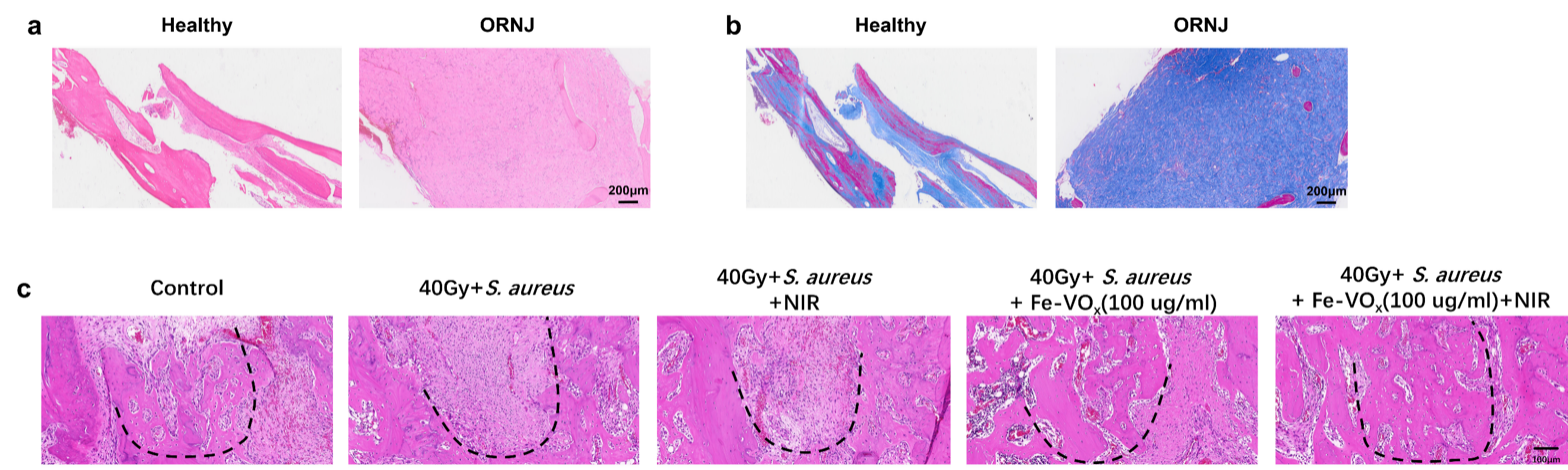
**

**Figure S32.** Overall pictures of **(a,b)** Figure S31 and **(c)**Figure 9d, scale bars, 100 μm or 200 μm.


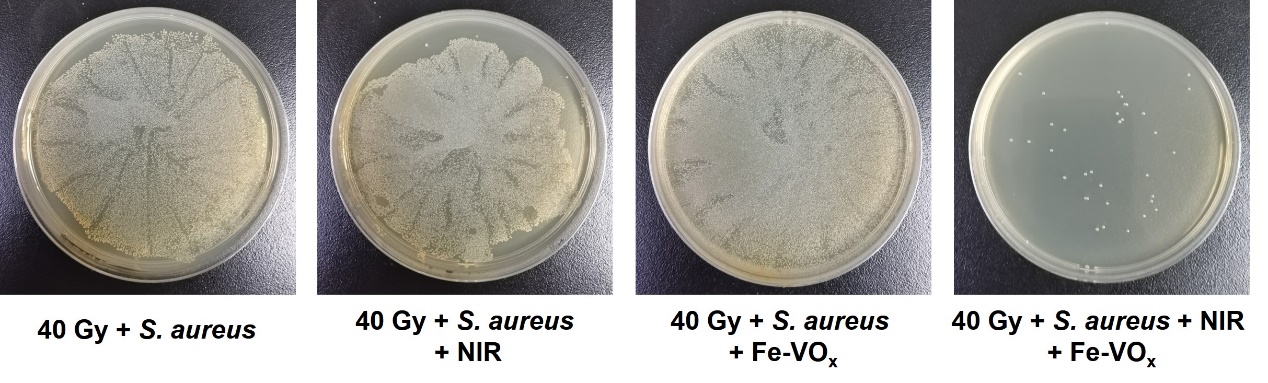


**Figure S33.** Colony counting formed by the bacteria collected from different treatment groups.


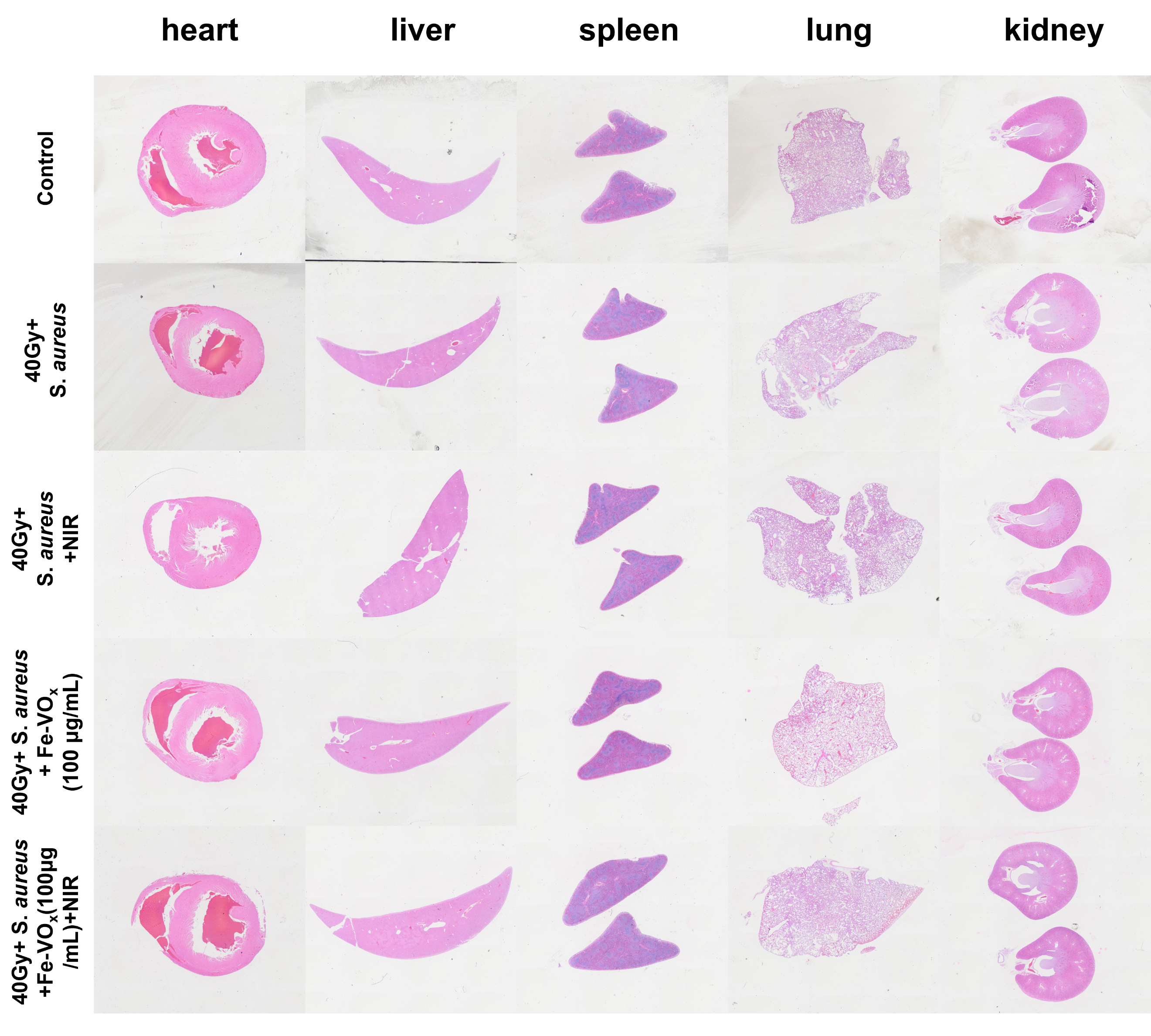


**Figure S34.** Observations of major organ tissue histomorphology heart, liver, spleen, lung, and kidney).


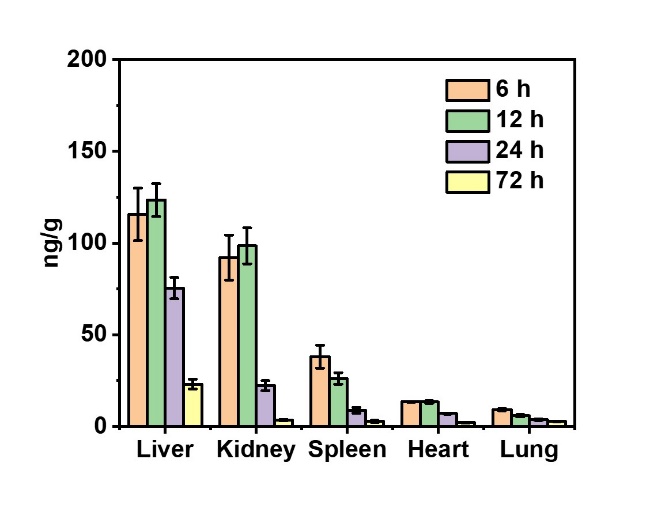


**Figure S35.** The biodistribution of V in main tissues in 6, 12, 24, and 72 h of administrations of Fe-VO_x_.


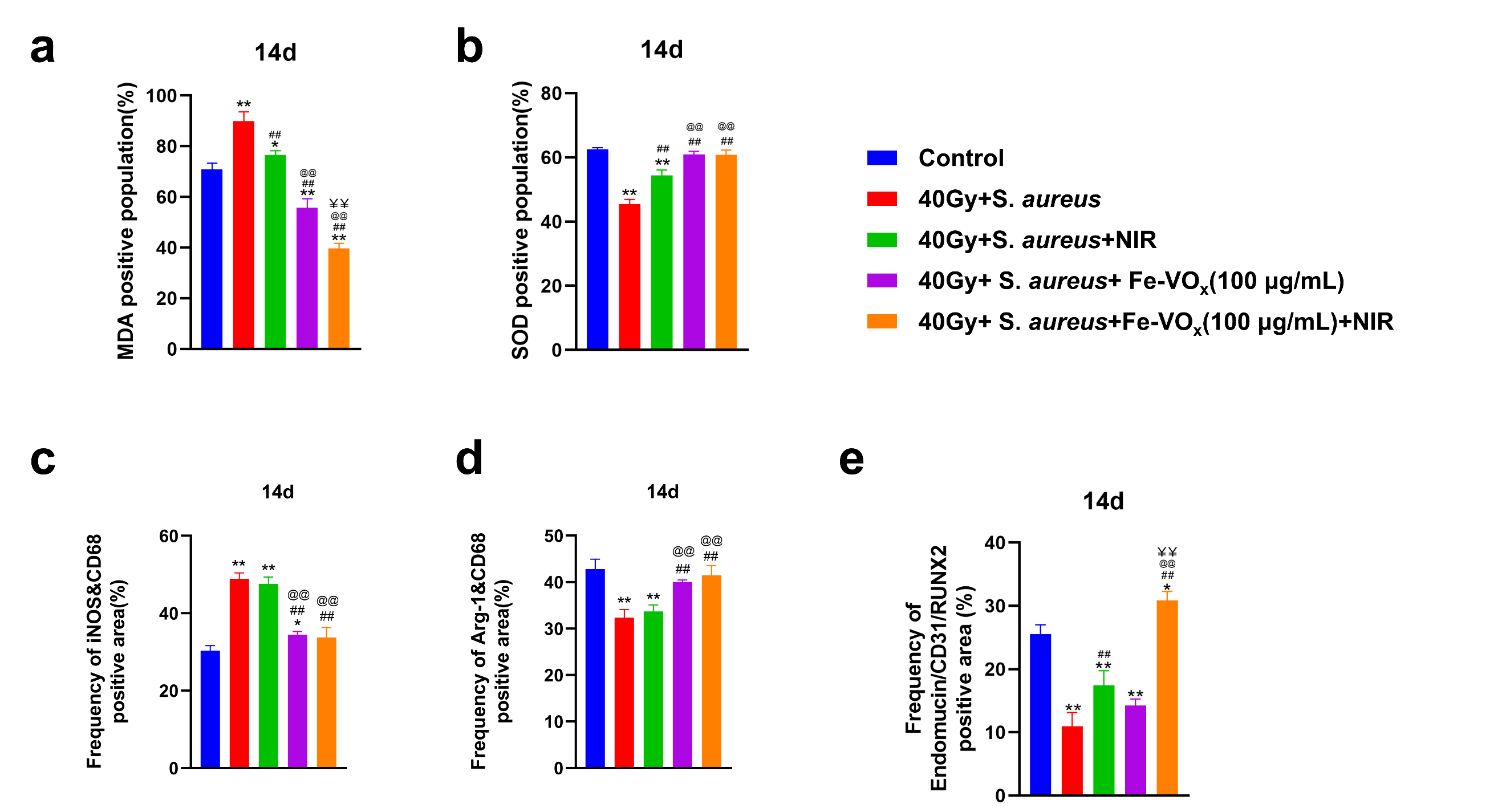


**Figure S36.** Quantification analysis of the frequency of **(a)** MDA, **(b)** SOD, **(c)** iNOS^+^/CD68^+^ colocalization, **(d)** Arg-1^+^/CD68^+^ colocalization and **(e)** CD31^+^/ Endomucin^+^/ RUNX2^+^ colocalization in TESs, respectively. ^**^P < 0.01 and ^*^P < 0.05 versus the control group, ^##^P < 0.01 versus the 40Gy+S. *aureus* group, ^@@^P < 0.01 versus the 40Gy+S. *aureus*+ NIR group, ^¥¥^P < 0.01 versus the 40Gy+ S. *aureus*+ Fe-VO_x_ (100 μg/mL) group. (n=3).


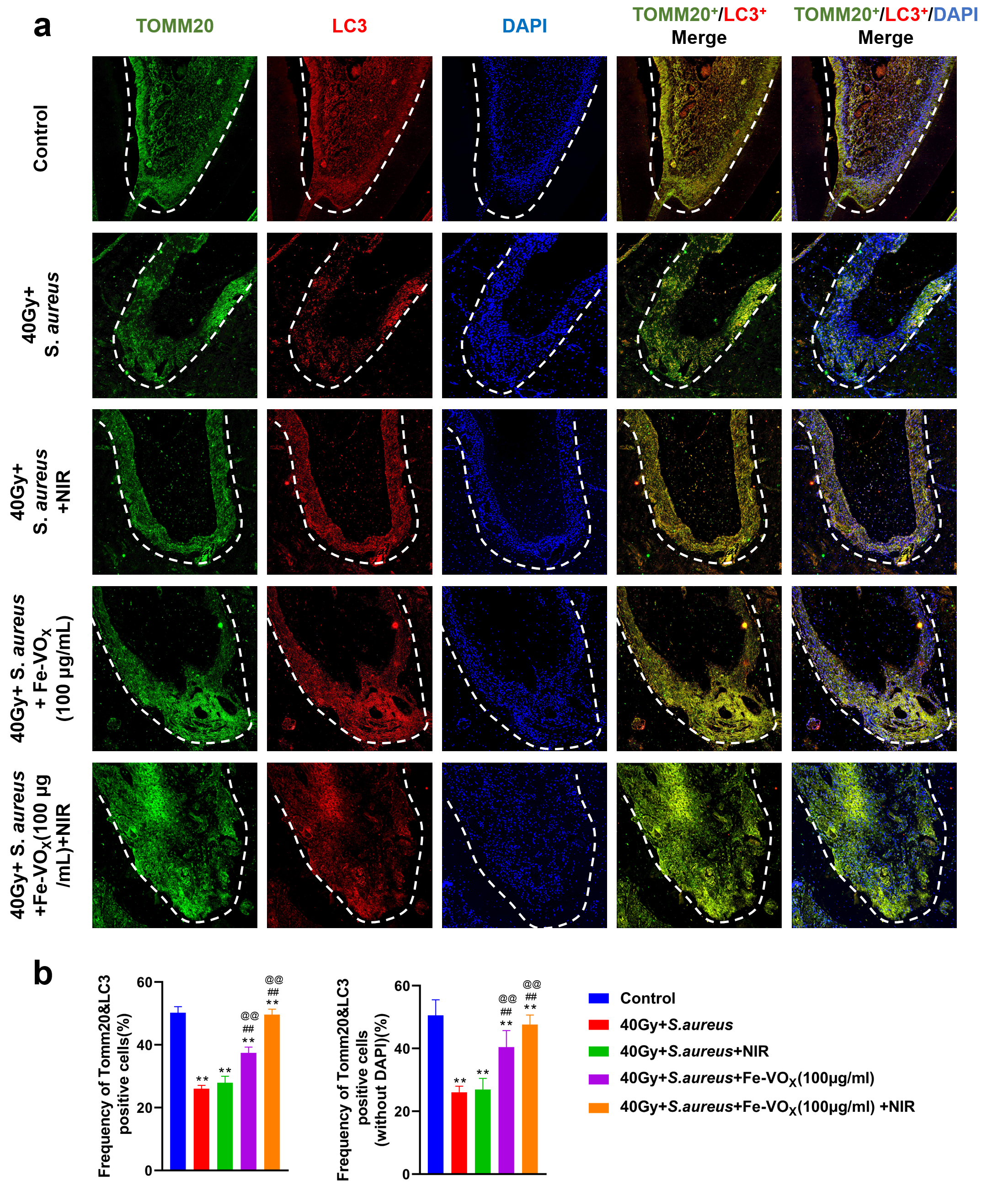


**Figure S37.** **(a)** Representative images and **(b)** quantification analysis of the frequency of TOMM20^+^/ LC3^+^ colocalization in TESs, respectively. ^**^P < 0.01 versus the control group, ^##^P < 0.01 versus the 40Gy+S. *aureus* group, ^@@^P < 0.01 versus the 40Gy+S. *aureus*+ NIR group. (n=3).


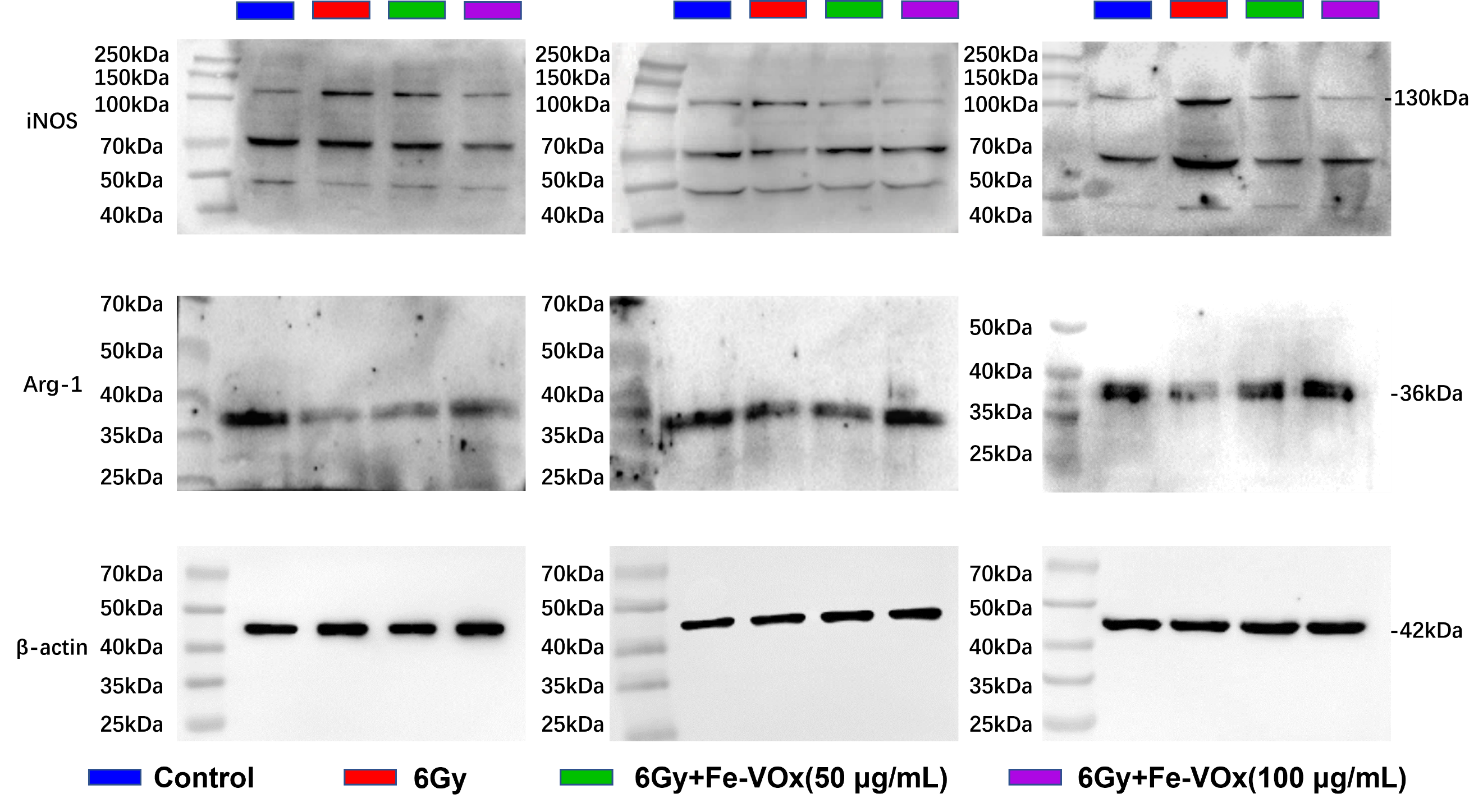


**Figure S38.** The images of uncropped blots for Figure 7d.


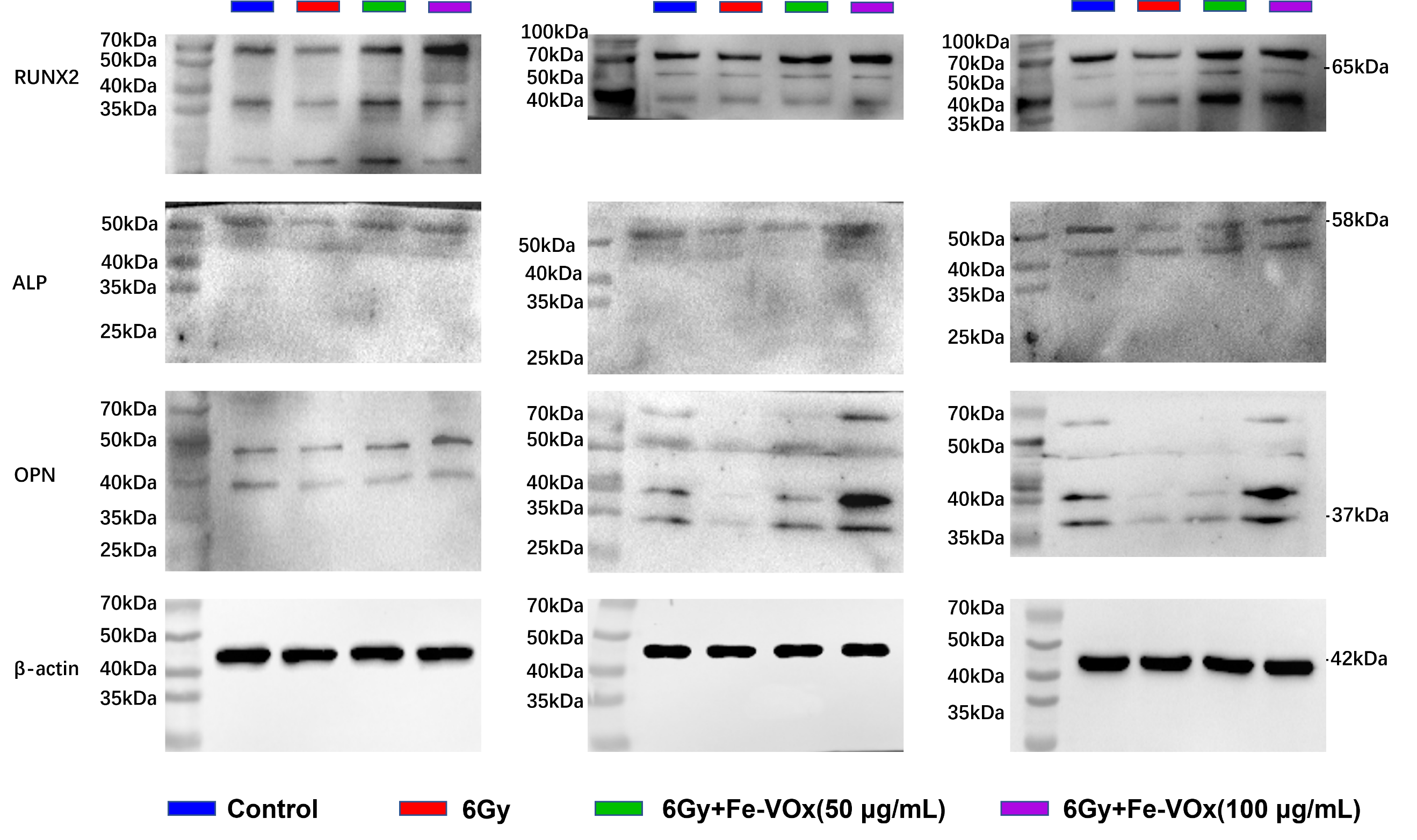


**Figure S39.** The images of uncropped blots for Figure 7f.


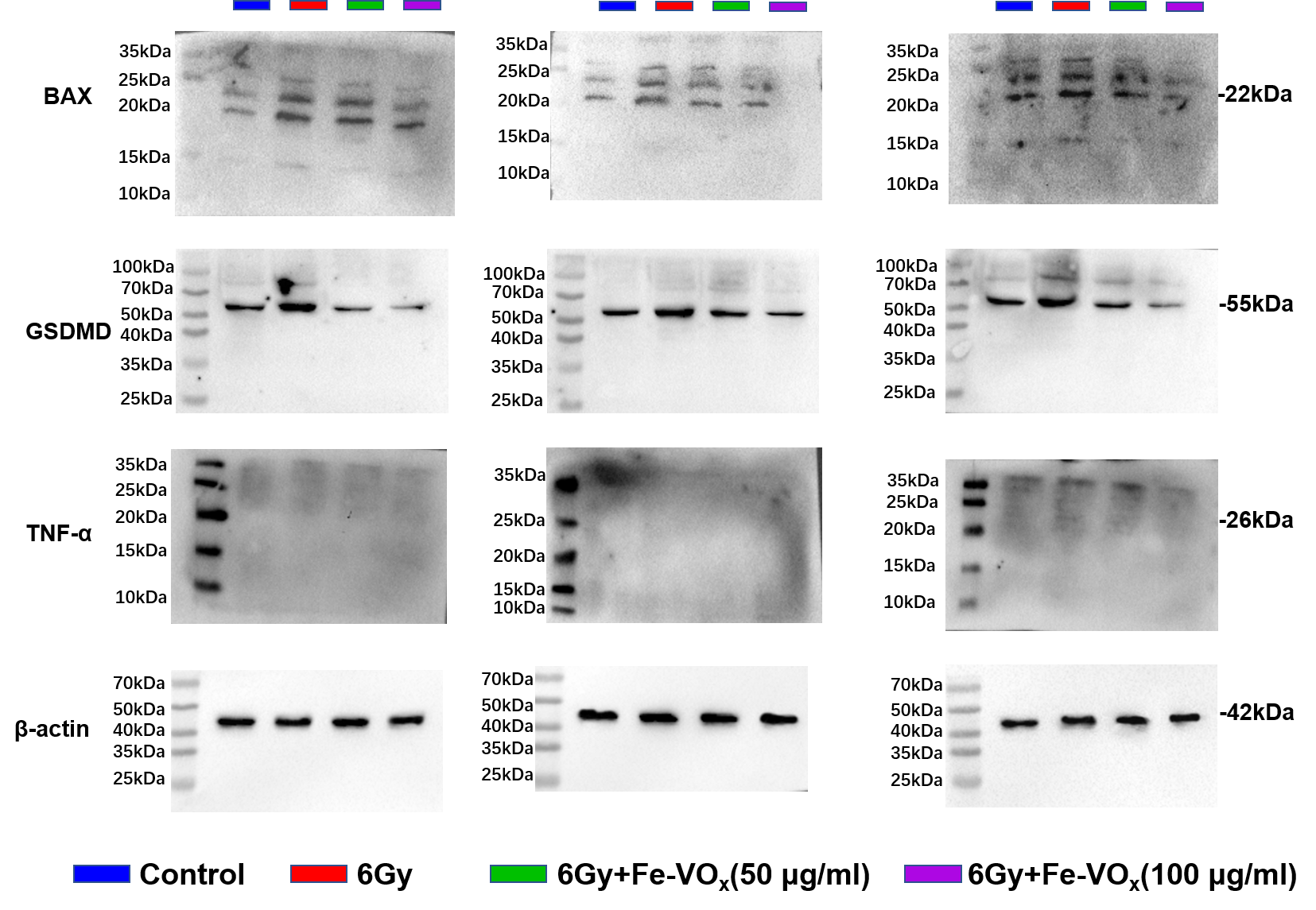


**Figure S40.** The images of uncropped blots for Figure S24e.

**Table S1.** The antibodies used for Western blotting

| **Antibody** | **Dilution** | **Source** | **Code number** |
| --- | --- | --- | --- |
| iNOS | 1:1500 | Proteintech | 18985-1-AP |
| Arg-1 | 1:1500 | Proteintech | 66129-1-Ig |
| ALP | 1:1000 | Origene | TA364673S |
| OPN | 1:1000 | Bioss | bs-0026R |
| RUNX2 | 1:1000 | Proteintech | 20700-1-AP |
| β-actin | 1:1000 | Bioworlde | BS6007MH |
| HRP Goat anti-rabbit lgG | 1:1500 | Proteinbio | PA2202 |
| BAX | 1:1000 | YpingBio | YP-Ab-00318 |
| GSDMD | 1:1000 | YpingBio | YP-Ab-12523 |
| TNF-α | 1:500 | Proteintech | 60291-1-Ig |

**Table S2.** The antibodies used for IF staining

| **Antibody** | **Dilution** | **Source** | **Code number** |
| --- | --- | --- | --- |
| DAF-FM | 1:1000 | Beyotime | S0019S |
| HPF | 1:1000 | Maokang Biotechnology | MX4805-1MG |
| ROS | 1:1000 | Beyotime | S0033S |
| SOD1 | 1:100 | affbiotech | AF5198 |
| IFIH1 | 1:200 | affbiotech | DF6926 |
| CD68 | 1:200 | boster | BA3638 |
| Arg-1 | 1:200 | PTG | 16001-1-ap |
| iNOS | 1:200 | Bioss | BS-0162R |
| CD31 | 1:1000 | abcam | ab182981 |
| Endomucin | 1:200 | bioss | BS-5884R |
| RUNX2 | 1:1000 | abcam | ab236639 |
| HSP60 | 1:250 | PTG | 15282-1-AP |
| LC3 | 1:250 | CST | 83406 |
| TOMM20 | 1:250 | abcam | ab221292 |
| LC3 | 1:250 | proteintech | 18725-1-AP |
| TOMM20 | 1:250 | proteintech | 11802-1-AP |
